# The Surfactin-Like Lipopeptides From *Bacillus* spp.: Natural Biodiversity and Synthetic Biology for a Broader Application Range

**DOI:** 10.3389/fbioe.2021.623701

**Published:** 2021-03-02

**Authors:** Ariane Théatre, Carolina Cano-Prieto, Marco Bartolini, Yoann Laurin, Magali Deleu, Joachim Niehren, Tarik Fida, Saïcha Gerbinet, Mohammad Alanjary, Marnix H. Medema, Angélique Léonard, Laurence Lins, Ana Arabolaza, Hugo Gramajo, Harald Gross, Philippe Jacques

**Affiliations:** ^1^Microbial Processes and Interactions, TERRA Teaching and Research Centre, Joint Research Unit BioEcoAgro, UMRt 1158, Gembloux Agro-Bio Tech, University of Liège, Avenue de la Faculté, Gembloux, Belgium; ^2^Department of Pharmaceutical Biology, Pharmaceutical Institute, Eberhard Karls Universität Tübingen, Tübingen, Germany; ^3^Laboratory of Physiology and Genetics of Actinomycetes, Instituto de Biología Molecular y Celular de Rosario (IBR-CONICET), Facultad de Ciencias, Bioquímicas y Farmacéuticas, Universidad Nacional de Rosario, Rosario, Argentina; ^4^Laboratoire de Biophysique Moléculaire aux Interfaces, TERRA Teaching and Research Centre, Joint Research Unit BioEcoAgro, UMRt 1158, Gembloux Agro-Bio Tech, Université de Liège, Gembloux, Belgium; ^5^Unité de Génie Enzymatique et Cellulaire UMR 7025 CNRS, Université de Picardie Jules Verne, Amiens, France; ^6^Inria Lille, and BioComputing Team of CRISTAL Lab (CNRS UMR 9189), Lille, France; ^7^Chemical Engineering, Products, Environment, and Processes, University of Liège, Liège, Belgium; ^8^Bioinformatics Group, Wageningen University, Wageningen, Netherlands

**Keywords:** surfactin, lipopeptide, *Bacillus* spp., biosurfactant, nonribosomal peptide

## Abstract

Surfactin is a lipoheptapeptide produced by several *Bacillus* species and identified for the first time in 1969. At first, the biosynthesis of this remarkable biosurfactant was described in this review. The peptide moiety of the surfactin is synthesized using huge multienzymatic proteins called NonRibosomal Peptide Synthetases. This mechanism is responsible for the peptide biodiversity of the members of the surfactin family. In addition, on the fatty acid side, fifteen different isoforms (from C12 to C17) can be incorporated so increasing the number of the surfactin-like biomolecules. The review also highlights the last development in metabolic modeling and engineering and in synthetic biology to direct surfactin biosynthesis but also to generate novel derivatives. This large set of different biomolecules leads to a broad spectrum of physico-chemical properties and biological activities. The last parts of the review summarized the numerous studies related to the production processes optimization as well as the approaches developed to increase the surfactin productivity of *Bacillus* cells taking into account the different steps of its biosynthesis from gene transcription to surfactin degradation in the culture medium.

## Introduction

Surfactin was firstly isolated in 1968 by Arima et al. as a new biologically active compound produced by *Bacillus* with surfactant activities, leading to its appellation. Its structure was elucidated firstly through its amino acid sequence (Kakinuma et al., 1969a) and then its fatty acid chain (Kakinuma et al., 1969b). Surfactin was thus characterized as a lipopeptide composed of a heptapeptide with the following sequence: L-Glu1-L-Leu2-D-Leu3-L-Val4-L-Asp5-D-Leu6-L-Leu7, forming a lactone ring structure with a β-hydroxy fatty acid chain. Bearing both, a hydrophilic peptide portion and a lipophilic fatty acid chain, surfactin is of amphiphilic nature, leading to exceptional biosurfactant activities and diverse biological activities.

Surfactins are actually considered as a family of lipopeptides, sharing common structural traits with a great structural diversity due to the type of amino acids in the peptide chain and the length and isomery of the lipidic chain (Ongena and Jacques, 2008). More than one thousand variants can potentially be naturally synthesized. This remarkable biodiversity mainly results from their biosynthetic mechanism.

This review is composed of 4 main sections. At first, a detailed description of the biosynthesis mechanisms will allow to understand origin of the biodiversity. Secondly, the diversity of variants will be seen, as well as its enhancement possibilities. Thirdly, the link between surfactin's varying structure and its properties and activities will be described. Lastly, the production process and its optimisation will be discussed, either for the whole surfactin family or for specific variants.

## Biosynthesis of Surfactins

### Peptide Moiety

**Figure 1:**
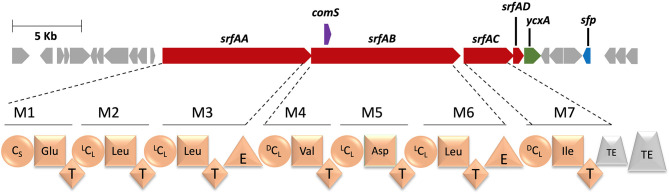
Top: The surfactin biosynthetic gene cluster. Structural NRPS genes are indicated in red. The regulatory gene comS, which is co-encoded in SrfAB is indicated in purple. Bottom: Classic module and domain architecture of SrfAA-SrfAD.

Surfactins, as most of the cyclic lipopeptides (CLPs), are not synthesized ribosomally, but rather by specialized systems, termed non-ribosomal peptide synthetases (NRPSs). NRPSs are multimodular mega-enzymes, consisting of repeated modules. A module is defined as a portion of the NRPS that incorporates one specific amino acid into a peptide backbone. The order of the modules is usually co-linear with the product peptide sequence. Each module can in turn be dissected into the following three domains: the adenylation (A) domain, the thiolation (T) domain (“-syn. peptidyl-carrier protein (PCP)-”) and the condensation (C) domain (Marahiel et al., 1997; Roongsawang et al., 2011). The A-domain recognizes, selects, and activates the specific amino acid of interest (Dieckmann et al., 1995). Taking into account the 3D-structures of several adenylation domains and their active site, several tools have been set up to correlate the amino acid residue present in this active site and their substrate specificity. A NRPS code was so defined that it is based on 8 amino acid residues from the active site (Stachelhaus et al., 1996; Rausch et al., 2005). The activated amino acid is hereby covalently bonded as a thioester to the flexible 4′-phosphopantetheinyl (4′-Ppant) arm of the T-domain. The 4′-Ppant prosthetic group is 20 Å in length and can swing from one to another adjacent catalytic center. Exactly this flexibility enables the transfer of the activated amino acid substrate to the C-domain, which catalyzes in turn (i) the formation of a peptide bond between the nascent peptide and the amino acid carried by the adjacent module and allows afterwards (ii) the translocation of the growing chain to the following module. Various functional subtypes of the C domain have been described. For example, an ^L^C_L_ domain catalyzes the formation of a peptide bond between two L-amino acids while a ^D^C_L_ domain between a L-amino acid and a growing peptide ending with a D-amino acid (Rausch et al., 2007). The first module (A-T module) is considered the initiation module, while the subsequent (C-A-T) modules are defined as elongation modules. After several module-mediated cycles of peptide extension, the complete linear intermediate peptide is released by the terminal thioesterase (TE) domain which, often, catalyzes an internal cyclization (Marahiel et al., 1997; Trauger et al., 2000). Besides the above mentioned domains, the NPRS assembly line can furthermore comprise additional optional domains, which catalyze modifications of amino acid building blocks e.g. their epimerization (E-domains) (Süssmuth and Mainz, 2017). The lipid moiety of surfactins and most of the microbial lipopeptides is introduced directly at the start of the biosynthesis. The initiation module features a C-A-T- instead of a classic A-T-structure (Sieber and Marahiel, 2005; Bloudoff and Schmeing, 2017). It contains a special N-terminal C-domain, termed C-starter (C_S_) domain and is in charge of the linkage of a CoA-activated β-hydroxy fatty acid to the first amino acid. The activated fatty acid stems foremost from the primary metabolism (Figure 1).

Three decades ago, the biosynthetic gene cluster (BGC) of the CLP surfactin was described in parallel by different research groups (Nakano et al., 1988; Cosmina et al., 1993; Fuma et al., 1993; Sinderen et al., 1993). The structural genes were identified in *B. subtilis* and are formed by the four biosynthetic core NRPS genes *srfAA, srfAB, srfAC*, and *srfAD* (Figure 1) which code together for a heptamodular NRPS assembly line. The three-modular enzyme SrfAA contains N-terminally the typical C_S_-domain of CLP-BGCs and acylates the first amino acid Glu1 with various 3-OH-fatty acids stemming from primary metabolism. The peptide is subsequently extended in a co-linear fashion by the elongation modules of SrfAA, SrfAB and SrfAC to yield a linear heptapeptide (FA-L-Glu1-L-Leu2-D-Leu3-L-Val4-L-Asp5-D-Leu6-L-Leu7). The inverted stereochemistry can be readily attributed to the presence of E-domains in modules M3 and M6 and ^D^C_L_ domains in modules M4 and M7 (Figure 1). Finally, the TE domain of SrfAC releases the lipopeptide and performs the macrocyclization between Leu7 and the hydroxy-group of the 3-OH fatty acid. Notably, SrfAD consist solely of a second TE-domain, which represents rather a supportive repair enzyme and is able to regenerate misprimed T-domains during NRPS assembly (Schneider et al., 1998; Schwarzer et al., 2002; Yeh et al., 2004).

Beside the structural NRPS genes, the surfactin BGC comprises one built-in and several adjacent accessory genes encoding e.g. transporters and regulatory proteins (MiBIG Accession No: BG0000433). Amongst these, we would like to further highlight the genes *sfp, ycxA, krsE, yerP* and *comS*, which are particularly related with the production yield of surfactin.

Sfp represents a phosphopantetheinyl transferase (PPTase) and is located ~4 kb downstream of the *srf* BGC. The T-domain of an NRPS is, upon its expression, not directly active but rather exists nascent in its non-functional apo-form. For full functionality, the flexible 4′-Ppant arm needs to be fused to the T-domain. The latter process is mediated by the PPTase Sfp, thereby converting all T-domains of the surfactin BGC into their active holo form (Quadri et al., 1998; Mootz et al., 2001). This fact makes Sfp indispensable for the production of surfactin (Tsuge et al., 1999). For example, in the reference strain, *Bacillus subtilis* 168, the *sfp* locus is truncated and therefore non-functional, which abolishes in turn surfactin production. However, the production can be restored by the transfer of a complete *sfp* locus (Nakano et al., 1988, 1992).

Further important genes in the context of surfactin production are genes encoding transporters which are efflux pumps. From a physiologically point of view, the pumps avoid intracellular surfactin accumulation and constitute an essential self-resistance mechanism (Tsuge et al., 2001). In particular since surfactin inserts into biomembranes and at higher concentration causes membrane disruption. An ecological rationale for transporters could be that surfactin is extracellularly at the correct site where it can exert its beneficial activity. So far, three transporters have been identified in Bacilli, that are involved in surfactin efflux, i.e. YcxA, KrsE, and YerP. It has been demonstrated that the separate overexpression of the corresponding genes enhanced release rates of surfactin (Li et al., 2015) by 89, 52, and 145%, respectively.

Finally, the surfactin BGC exhibits a unique peculiarity on the genetic level, in bearing a co-encoded regulatory gene, termed *comS* inside itself (D'Souza et al., 1994). It is located in the open reading frame of the NRPS gene *srfAB* (Hamoen et al., 1995), more precisely within the A-domain of module 4 (Figure 1). ComS is on the one hand involved in the positive regulation of the genetic competence of the cell (Liu and Zuber, 1998) and on the other hand part of the quorum sensing system *comQXPA* (Ansaldi et al., 2002; Schneider et al., 2002; Auchtung et al., 2006) which in turn regulates surfactin production. Beyond this brief explanation, for an excellent overview about the role of ComS, the reader is referred to a review, written by Stiegelmeyer and Giddings (2013). Since the production yield is coupled with the presence and functionality of ComS in the coding region of *srfAB*, the genetic engineering of the surfactin synthetase in this region requires special attention.

### Fatty Acid Chain Synthesis

Since fatty acid biosynthesis plays a critical role in surfactin production, and strongly determines its activity and properties, in this section we briefly summarize this central metabolic pathway and the subsequent steps leading to the modification and activation of the fatty acyl-CoA precursor.

**Figure 2:**
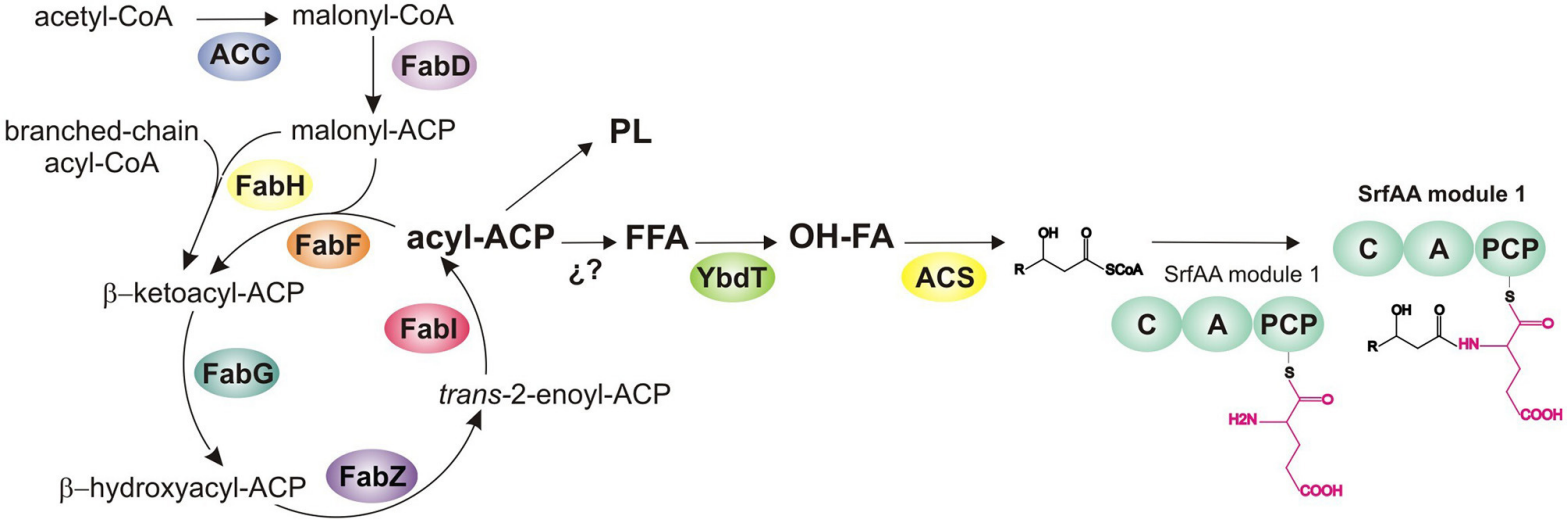
Biochemical steps for the formation of fatty acid and their channeling to surfactin biosynthesis. The first step of fatty acid synthesis involves the production of malonyl-CoA by the acetyl-CoA carboxylase complex (ACC). The malonyl-CoA-ACP transacylase, FadD, transfers the malonyl groups to the acyl carrier protein (ACP) to produce malonyl-ACP. FabH, condensates the malonyl-ACP and a priming acyl-CoA substrate to produce the first new C-C bond. The keto group of the β-ketoacyl-ACP is completely reduced by the reducing enzymes of the cycle, FabG, FabZ, FabI, and then the condensing enzyme FabF initiates a new round of elongation of the growing carbon chain utilizing malonyl-ACP. The acyl-ACP product is primarily channeled to PL biosynthesis or alternatively to surfactin biosynthesis. For this, at least two additional biochemical steps are required, a hydroxylation of a free FA by YbdT and its activation by an ACS.

All organisms employ a conserved set of chemical reactions to achieve the *de novo* Fatty Acid (FA) biosynthesis, which works by the sequential extension of the growing carbon chain, two carbons at a time, through a series of decarboxylative condensation reactions (Wakil et al., 1983) (Figure 2). This biosynthetic route proceeds in two stages: initiation and iterative cyclic elongation. The acetyl-CoA carboxylase enzyme complex (ACC) performs the first committed step in bacterial FA synthesis to generate malonyl-CoA through the carboxylation of acetyl-CoA (Marini et al., 1995; Tong, 2013). The malonate group from malonyl-CoA is transferred to the acyl carrier protein (ACP) by a malonyl-CoA:ACP transacylase (FabD) (Serre et al., 1994, 1995; Morbidoni et al., 1996). The first reaction for the synthesis of the nascent carbon chain comprises the condensation of malonyl-ACP with a short-chain acyl-CoA (C2–C5) catalyzed by a 3-keto-acyl carrier protein synthase III (FabH). Acetyl-CoA is used as a substrate for the synthesis of straight-chain FA, while branched-chain fatty acids (BCFA) arise from isobutyryl-CoA, isovaleryl-CoA and methylbutyryl-CoA priming substrates. These precursors derive, from the catabolism of the branched-chain amino acids valine, leucine and isoleucine, respectively. The crucial branched-chain α-keto acid decarboxylase (BKD) complex catalyzes the decarboxylation of α-keto acids to generate the corresponding branched-chain acyl-CoA primers (Willecke and Pardee, 1971; Kaneda, 1991; Lu et al., 2004). The substrate specificity of FabH plays a determining role in the branched/straight and even/odd characteristics of the fatty acid produced. *B. subtilis* possesses two FabH isoenzymes, FabHA and FabHB, both of which preferentially utilize branched-chain acyl-CoA primers (Choi et al., 2000). Therefore, BCFA are the main components of phospholipids, where iso-C15:0, anteiso-C15:0, iso-C16:0, iso-C17:0, and anteiso-C17:0 represent the major FA found in *Bacillus* species (Kaneda, 1969; Kämpfer, 1994). The pattern of the BCFA can be modified by environmental conditions such as temperature (Graumann and Marahiel, 1999).

Next, the keto-acyl-ACP product of FabH condensation enters the elongation/reducing cycle of the fatty acid synthase II (FAS-II). There, the keto group is reduced by the NADPH dependent β-ketoacyl-ACP reductase (FabG) to give β-hydroxy-acyl-ACP. The β-hydroxyacyl-ACP intermediate is then dehydrated to *trans*-2-enoyl-ACP by a 3-hydroxyacyl-ACP dehydratase (FabZ). Then, the cycle is completed by an enoyl-ACP reductase, which reduces the double bond in *trans*-2-enoyl-ACP to form acyl-ACP (Fujita et al., 2007). *B. subtilis* possesses two enoyl-ACP reductases (FabI and FabL) with opposite preferences for the NADPH or NADH cofactor (Heath et al., 2000).

In all the successive steps of FA elongation, the acyl-ACP intermediate and malonyl-ACP are the substrates of FabF condensing enzyme (3-oxoacyl-ACP-synthase II) that elongates the growing acyl chain and initiate each new round of the cycle (Schujman et al., 2001). Finally, the acyl-ACPs of the proper chain length are substrates of acyltransferases involved in cell membrane phospholipid synthesis. Alternatively, some structurally specific FA are not integrated in the cell membrane phospholipids. Those modified FA could be, under specific environmental or growth conditions, channeled into secondary metabolic pathways. They are then a of specialized molecules, as it is the case of lipopeptides.

Once the long chain FA is synthesized, the next steps needed for surfactin biosynthesis involves the production of the 3-hydroxy-acyl-coenzyme A (CoA) substrates. Youssef et al. based on *in vitro* assays, suggested that acyl 3-hydroxylation occurs prior to CoA ligation (Youssef et al., 2011). These authors reported that YbdT, a cytochrome P450 enzyme, catalyzes the hydroxylation of the FA precursors to be incorporated in the lipopeptide biosynthetic pathway (Youssef et al., 2011). Cytochrome P450 are monooxigenases capable of introducing an oxygen atom into FA and in other lipidic and non-lipidic molecules. The *B. subtilis* genome contains eight genes coding for cytochrome P450 enzymes (Hlavica and Lehnerer, 2010). *In vitro*, high-performance liquid chromatography (HPLC) and gas chromatography–mass spectrometry analyses demonstrated that the recombinant *ybdT* gene product hydroxylates myristic acid in the presence of H_2_O_2_, to produce β-hydroxymyristic acid and α-hydroxymyristic acid (Matsunaga et al., 1999). Furthermore, a *ybdT* mutant strain of *B. subtilis* OKB105 produces biosurfactants with only 2.2% of 3-hydroxylated C14, while the 97.8% contained non-hydroxylated FA with chain lengths of C12, and C14–C18 (Youssef et al., 2011) and are thus linear.

Finally, the surfactin synthetase assembly line can be initiated in presence of a CoA-activated FA (Steller et al., 2004). Fatty acids are converted into their corresponding acyl-CoA derivative by fatty acyl CoA ligases (FACS). Of the four putative FACS identified in homology searches in the genome of *B. subtilis*, two of them, LcfA and YhfL, were characterized *in vitro* to be involved in surfactin production. HPLC-MS based FACS activity assays indicated that LcfA and YhfL catalyze the thioester formation with CoA and various FA substrates (3-OH C8, 3-OH C10, C12, and C14). All four single mutants in the FACS homolog genes, *lcfA, yhfL, yhfT* and *yngI*, decreased surfactin production by 38% - 55%, compared with the wild-type levels. Interestingly, a quadruple mutant in the FACS did not completely abolish surfactin biosynthesis, such strain still presents 16% surfactin production, compared with the levels produced by the wild-type strain. This observation suggests that other non-canonical FACS are present in *B. subtilis* or that other pathways, such as transthiolation from ACPs to CoA, could be involved in providing the fatty acyl moiety.

The hydroxylated and CoA activated FA derivative is finally transferred onto the surfactin synthetase assembly line, in a reaction performed by the N-terminal condensation (C_S_) domain, that is as mentioned above responsible for the lipoinitiation mechanism. *In vitro*, the recombinant dissected C domain, catalyzed the acylation reaction using glutamate-loaded PCP domain and 3-OH-C14-CoA as substrates (Kraas et al., 2010).

## Variants of Surfactin

The surfactin biosynthesis mechanism previously described is responsible for the high biodiversity of surfactin-like molecules. In addition, the assembly line machinery of surfactin synthetases can be easily modified by synthetic biology in order to increase this biodiversity. Both aspects will be developed in the following chapter.

### Natural Variants

**Figure 3:**
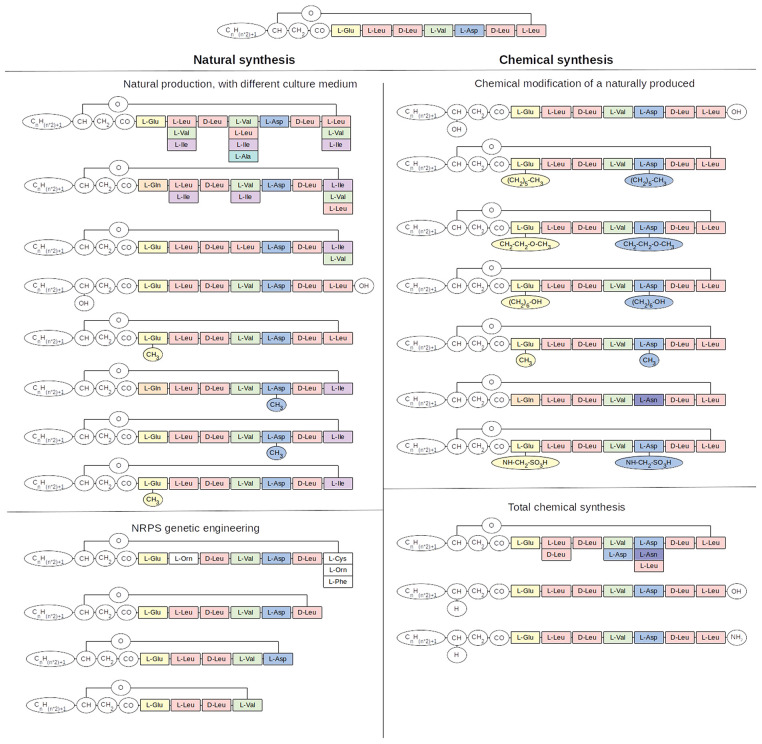
Natural and synthetic variants of surfactin. The natural variants can be obtained through specific strains, the non specificity of the adenylation domain or the first condensation domain, a non cyclization or a linearization and through the genetic engineering of the NRPS. The synthetic variants can be obtained through a chemical modification of a natural product or through total chemical synthesis. The first three molecule naturally produced are surfactin produced by B. subtilis and others, pumilacidin from B. pumilus and lichenysin from B. licheniformis.

Three main peptide backbones and the NRPSs responsible for their biosynthesis, produced by different *Bacillus* species, have been so far described in literature: surfactin as previously described from *B. subtilis, B. amyloliquefaciens, B. velezensi*, and *B. spizizeni* amongst others, pumilacidin from *B. pumilus* (Naruse et al., 1990) and lichenysin from *B. licheniformis* (Horowitz et al., 1990). Compared to surfactin, pumilacidin has a leucine in position 4 instead of a valine, as well as an isoleucine or a valine in position 7 instead of a leucine. Lichenysin differs from surfactin by a change in the first amino acid residue: a glutamine (Gln) instead of a glutamic acid (Figure 3).

This first biosynthetic diversity in surfactin is increased by the promiscuous specificity of adenylation domains of modules 2, 4, and 7 of surfactin synthetases which are able to accept L-Leu, L-Val or L-Ile amino acids residues as well as L-Ala for module 4. Similarly low levels of specificity have been observed for lichenysin (Peypoux et al., 1991; Bonmatin et al., 2003).

Based on all these results, it appears that the aspartic acid in position 5, as well as the D-Leucine in position 3 and 6 are present in all the members of the surfactin family. The only mention of an asparagine (Asn) for lichenysin (Yakimov et al., 1995) was quickly refuted by the same author after the use of fast atom bombardment mass spectrometry (Yakimov et al., 1999). The specificity of M3 and M6 could result from (i) an enzyme of the assembly line machinery such as the epimerisation domain which could accept only leucine as substrate, (ii) from the specificity of the adenylation domain or (iii) from the specificity of the involved condensation domains.

The changes in the peptide chain are not the only source of diversity in the surfactin family. As mentioned before, surfactin is a heptapeptide linked to a fatty acid chain. Regarding this chain, the length of it can vary from 12 to 17 carbons atoms, mainly being C14 and C15.

Another change in this lipid chain is its isomery, it can have a linear, *n*, configuration, but it can also be branched, iso and anteiso. Anteiso can only be in an uneven carbon chain length, while iso can be found in all chain lengths (odd and even-numbered carbon chain). These derivatives can be mainly explained by the promiscuity of the C_S_-domain present in module M1 toward its relaxed substrate specificity.

Finally, natural linear surfactins (Figure 3) have been also identified in the culture supernatant of *Bacillus* strains (Gao et al., 2017). The molecular mechanism responsible for this linearization is not yet known. It could result from an incomplete efficacy of TE domain which could release some surfactin without cyclization or from enzymatic or chemical degradation of cyclic surfactin.

In addition, heterologous enzymes are also capable to catalyze linearization. An *in vitro* study showed the linearisation effect of a purified V8 endoprotease from *Staphylococcus aureus* (Grangemard et al., 1999). Furthermore, an *in vivo* study demonstrated that *Streptomyces* sp. Mg1 produces, as a mechanism of resistance, an enzyme that hydrolyses surfactin into its linear form (Hoefler et al., 2012).

Surfactin methyl ester was observed in the supernatant of Ba*cillus subtilis* HSO121 (Liu et al., 2009), and a methylated product of surfactin with a valine in position 7 was discovered in the supernatant of a *Bacillus* mangrove bacteria strain (Tang et al., 2007). This change was also discovered in the supernatant of *Bacillus licheniformis* HSN221 with surfactin and lichenysin methyl esters (Li et al., 2010) and in the culture medium of *Bacillus pumilus* through surfactin methyl ester (Zhuravleva et al., 2010).

### Synthetic and Biosynthetic Variants

In addition to the natural surfactins seen before, synthetic variants can be obtained through chemical modifications or genetic engineering of the NRPS. This leads to new forms or to a controlled production of a specific form. Reasons for structural changes are manifoldly given, foremost to reduce the toxicity of surfactin, but also to optimize its biological activities or to increase its water solubility.

Esterification can be achieved through chemical treatment with alcohol, reacting with the Asp-β- and/or Glu-γ-carboxyl group, producing monoester, and/or diester-surfactin (Figure 3).

For example, reaction of surfactin with *n*-hexyl alcohol lead to mono- and *di-*hexyl-surfactin, with 2-methoxyethanol to mono- and *di-*2-methoxy-ethyl-surfactin (Shao et al., 2015). Amidation through a reaction with alcohol and then NH_4_Cl was also observed (Morikawa et al., 2000). Esterification and amidation of aspartic and glutamic acid eliminate the negative charge of those amino acid residues, creating an even greater diversity in the surfactin family because of the charge change that they bring and thus the modification in surfactin biological and surfactant properties.

Linearization of the cyclic surfactin previously mentioned as a natural process can also be obtained by chemical alkaline treatment (Figure 3) (Eeman et al., 2006).

In addition to those chemical modifications of surfactin naturally produced, synthetic forms can be chemically produced (Figure 3). Liquid phase techniques have been used at first (Nagai et al., 1996) but, because of the many steps and the purification of intermediates needed, it was replaced with a quicker solid phase peptide synthesis (SPPS) technique. Different forms of surfactins have been produced, such as standard surfactin, but also analogs with a change in the amino acid sequence, such as an epimerisation (D-Leu2), a change in charge (Asn5) and the switch of two residues (Asp4-Leu5) (Pagadoy et al., 2005). Linear surfactin was also produced, as well as linear with an amidated carboxy-terminus function (Dufour et al., 2005). Finally, the fatty acid chain length was likewise changed, with C10 and C18 (Francius et al., 2008). However, due to the complexity of the production, these lipopeptides are intended only for research use.

As said before, in addition to the chemical changes, the genetic engineering can be also applied to the genes coding for the NRPS, in order to modify the structure of surfactin. The generation of novel derivatives by rational design can hereby be achieved by site directed mutagenesis, module- insertion, deletion, and substitution (Alanjary et al., 2019). Application of the site directed mutagenesis technique, an A-domain specificity of an NRPS module shift from L-Glu to L-Gln and from L-Asp to L-Asn at position 5 in modules 1 and 5 was accomplished, respectively (Eppelmann et al., 2002).

Concerning the concept of module substitutions, particularly the Marahiel group showed in a ground breaking way from the mid 90s onwards the feasibility of module swaps which allowed single or multiple variations concerning all seven amino acids (Stachelhaus et al., 1995, 1996; Schneider et al., 1998; Eppelmann et al., 2002). As a practical aspect, beside the gain in basic research knowledge, for several modified surfactins, such as Cys7-surfactin, a decreased hemolytic activity was observed. Furthermore, ring contracted surfactin derivatives were obtained by deletion of complete NRPS modules. In this way, the corresponding knockouts yielded hexapeptidic surfactin congeners, individually lacking Leu2, Leu3, Asp5 and Leu6. Notably, the ΔLeu2 ΔLeu3 and the ΔLeu6 surfactin variants showed a reduced toxicity toward erythrocytes and enhanced antibacterial activities, while the ΔAsp5 surfactin exhibited an even higher inhibitory ability for Gram positive bacteria, but kept the hemolytic capabilities of the native surfactin (Mootz et al., 2002; Jiang et al., 2016). However, each genetic manipulation mentioned above resulted in a significant decrease in the production yield. Nevertheless, these studies showed the feasibility and moreover demonstrated in an encouraging way that the surfactin scaffold can be fine-tuned concerning its intended activity and its undesired side effects.

**Figure 4:**
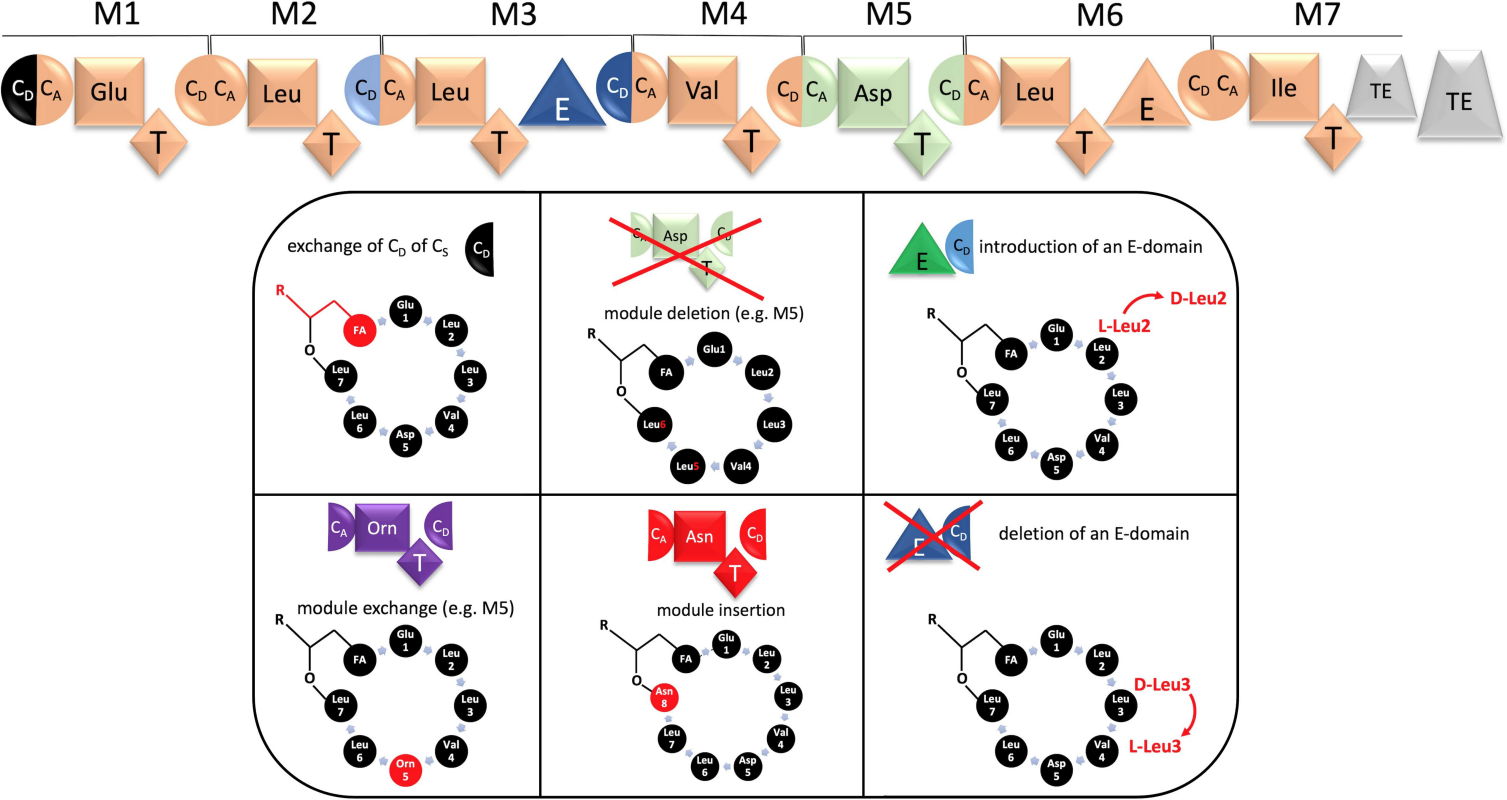
Top: Re-defined module and domain architecture of SrfAA-SrfAD with dissected C subdomains. The new module definition C_A_-A-T-C_D_ is indicated in light green. BOTTOM: Examples of biobrick-like exchanges and deletions using a synthetic biology concept. The resultant changes in the molecule are indicated in red. R represents the rest of the fatty acid moiety, which has numerous possibilities regarding chain length, degree of saturation and branching.

Very recently, the Bode group revolutionized the concept of module swapping. It includes the finding that C-domains have to be subdivided into a C_Donor_ (C_D_) and C_Acceptor_ (C_A_) portion and that both are amino-acid specific (Bozhüyük et al., 2019). This redefines nowadays the borders of an exchange unit. Instead of a classic A, A-T or C-A-T domain swap, it is preferable to exchange a C_D_-A-T-C_A_ domain unit (Figure 4). The huge advantage of these findings is that peptide-variants can be generated by genetic engineering at a much higher success rate and without any production loss. The technique will be an incentive to modify highly bioactive structures, such as surfactin. The exchange units can be derived from other Bacilli or codon-optimized from other bacterial genera. Particularly, in combination with synthetic biology, in future numerous genetically-engineered modifications can be envisioned: beside the exchange of amino acids, ring contractions by module deletion and ring expansions, by addition of an exchange unit, can be generated, respectively (Figure 4). Since peptides, containing D-configured amino acids are less prone to degradation, the change of the absolute configuration by insertion of epimerization domains could lead to derivatives that are less prone to enzymatic degradation. Furthermore, since the biotechnological production of surfactin always results in the production of complex mixtures, e.g., varying in the fatty acid portion, it would be desirable to produce surfactin with a more defined lipid moiety. For this purpose, the biobrick-like exchange of the C_Donor_-portion of the C_S_-domain could lead to the incorporation of the desired 3-OH fatty acid. Finally, it can be also envisioned to modify the surfactin NRPS assembly line even further, e.g. by introduction of catalytic domains which drive intramolecular cyclization-, N-methylation-, hydroxylation-, and redox-reactions.

## Structure and Properties Relationship

Surfactins and surfactin-like molecules are amphiphilic molecules with a polar part mainly constituted by the two negatively charged amino acid residues Glu and Asp (in native surfactin) and an apolar domain formed by the lateral groups of aliphatic amino acid residues (mainly Leu) and the fatty acid chain. This amphiphilic structure is responsible for its attractive physico-chemical properties as well as its various biological activities.

### Surfactin Structure and Its Influence on Physico-Chemical Properties and Biological Activites

The amphiphilic structure of surfactins leads to strong surface activity, i.e., their capacity to reduce the surface/interfacial tension and to self-assembly in nanostructures, and the presence of negative charge(s). Thus, they display as physico-chemical properties foaming (Razafindralambo et al., 1998; Fei et al., 2020), emulsifying (Deleu et al., 1999; Liu et al., 2015; Long et al., 2017; Fei et al., 2020) and dispersing properties, solid surface wetting and surface hydrophobicity modification performance (Ahimou et al., 2000; Shakerifard et al., 2009; Marcelino et al., 2019; Fei et al., 2020), and chelating ability (Mulligan et al., 1999; Grangemard et al., 2001; Eivazihollagh et al., 2019). This strong surface activity leads to detergent applications (Zezzi do Valle Gomes and Nitschke, 2012), but they also show promising perspectives of applications in the environmental sector to enhance oil recovery in oil-producing wells (Liu et al., 2015; Joshi et al., 2016; Long et al., 2017; de Araujo et al., 2019; Alvarez et al., 2020; Miyazaki et al., 2020), to increase the biodegradation rate of linear and aromatic hydrocarbons (Wang et al., 2020), and for metal removal from soil or aqueous solutions (Zouboulis et al., 2003; Eivazihollagh et al., 2019). Very recently, it was also suggested that surfactin can effectively demulsify waste crude oil (Yang et al., 2020). Their emulsifying property also confers them a potential of application in the food and cosmetics area for the product formulation (Mnif et al., 2013; Varvaresou and Iakovou, 2015; Zouari et al., 2016) as well as in the pharmaceutical area for the formulation of stable microemulsion drug delivery systems (Ohadi et al., 2020).

The variations in the molecular structure of the peptidic part and/or of the hydrocarbon chain greatly impact their physico-chemical properties. In term of self-aggregation behavior, the critical micellar concentration (CMC) value decreases with a longer fatty acid chain (CMC Surfactin C15 = 20 μM; CMC surfactin C14 = 65 μM; CMC surfactin C13 = 84 μM in Tris-HCl pH 8) (Deleu et al., 2003; Liu et al., 2015). It also decreases with the presence of a methyl ester on the Glu residue (Grangemard et al., 2001) or the replacing of the Glu residue by a Gln as in lichenysin (Grangemard et al., 2001; Bonmatin et al., 2003). On the contrary, the linearization of the peptide cycle (CMC linear surfactin C14 = 374 μM in Tris pH 8.5) (Dufour et al., 2005) and the presence of a Leu4 instead of the Val4 as in pumilacidin (de Araujo et al., 2019) increase it. Different self-assembled nanostructures like sphere-like micelles, wormlike micelles and unilamellar bilayers coexist with larger aggregates in aqueous solution depending on the surfactin concentration, pH, temperature, ionic strength and metal ions (Zou et al., 2010; Taira et al., 2017; Jahan et al., 2020). These parameters can induce conformational changes in the secondary structure of the cyclic peptide moiety and thereby affect the shape and the packing parameter of surfactin (Jahan et al., 2020).

The capacity of surface tension reducing is also influenced by the molecular structure of surfactin. Depending of environmental conditions, lichenysin is or not more efficient than surfactin to reduce the surface tension (in Tris pH 9.4 γ_cmc_=35 and 37 for lichenysin and surfactin respectively and in NaHCO_3_ pH 9.4 γ_cmc_=30 and 29 for lichenysin and surfactin respectively) (Grangemard et al., 2001), while pumilacidin is less (de Araujo et al., 2019). Linearization of the peptide cycle lessens this capacity (34 mN/m in Tris pH 8.5). Nevertheless, the replacing of carboxyl group by a sulfo methylene amido group leads to a complete loss of activity (Bonmatin et al., 2003). The chain length but also the branching type also impact the surface tension. A longer chain is more efficient and the normal configuration is more active than the iso one which is more powerful than the anteiso (Yakimov et al., 1996).

The effect of the chain length on the foaming properties does not follow this trend as it was shown that a lipidic chain with 14 carbon atoms provides surfactin with best foaming properties compared to that with 13 or 15 carbon atoms (Razafindralambo et al., 1998).

Lichenysin was also demonstrated to be a better divalent cation chelating agent than surfactin (Grangemard et al., 2001). This effect is assigned to an increase accessibility of the carboxyl group to the cation in the case of lichenysin (Habe et al., 2018). The complexation of divalent cations with the lipopeptide in a molar ratio of 2:1 for lichenysin leads to the formation of an intermolecular salt bridge, stronger than the intramolecular complexation in a 1:1 ratio with surfactin (Grangemard et al., 2001; Habe et al., 2018).

Globally speaking, the few studies focused on the structure-properties relationships of surfactin family emphasize three main facts. The first is that the unique feature of the peptide loop provides surfactin with a fascinating molecular behavior at interfaces (Liu et al., 2020). Furthermore, the peptide cycle linearization leads to a structural distortion of the molecule reducing or annihilating its surface active power. The second fact is that the surface activity of surfactin is dictated by the interplay of hydrocarbon chain and peptide sequence (Liu et al., 2020). The more distant and distinct the polar and apolar domains are, the stronger the surface active power is. The last fact is that the charges of the polar part also play a primordial role in the physico-chemical properties. A monoanionic surfactin is more efficient than a dianionic one, due to a reduced repulsive effect between the molecules at the interface.

The remarkable physico-chemical properties of surfactin are also responsible for their biological activities which, in most of the cases, involve perturbation or disruption of membrane integrity. It was demonstrated for haemolytic (Kracht et al., 1999; Dufour et al., 2005), antibacterial (Bernheimer and Avigad, 1970), antiviral (Yuan et al., 2018; Johnson et al., 2019), and antimycoplasma (Vollenbroich et al., 1997) activities of surfactin as well as its ability to inducing systemic resistance in plant (Ongena et al., 2007; Ongena and Jacques, 2008). Some of those activities leading to promising results in the agricultural field (Chandler et al., 2015; Loiseau et al., 2015). But surfactin was also characterized for anti-inflammation (Takahashi et al., 2006; Zhao et al., 2017), anti-sepsis (Hwang et al., 2007), anti-tumor (Wu et al., 2017) and immunomodulatory (Park and Kim, 2009) activities for which another target than membranes is involved. A synergistic effect has been observed between surfactin and other lipopeptides. The addition of surfactin at an inactive concentration to iturin increase its haemolytic activity (Maget-Dana et al., 1992). The combination of surfactin and fengycin lead to a decrease in disease in tomato and bean plants (Ongena et al., 2007). Furthermore, while surfactin has no effect against fungi, it has been shown to enhance the biological activities of other lipopeptides against fungi and oomycetes (Deravel et al., 2014; Tanaka et al., 2015; Desmyttere et al., 2019).

### Use of Molecular Modeling for Mechanism of Action Investigation

Molecular modeling methods are powerful theoretical tools to investigate structure functions relationship of surfactin and its mode of action. Docking and Molecular Dynamic (MD) simulations have been used in various studies involving surfactin for the characterization of diverse properties to predict activities and domains of applications.

For membrane interactions, Hypermatrix (Brasseur et al., 1987), was used to simulate the interaction of surfactin with a membrane monolayer in order to determine the lipid specificity for insertion and membrane destabilization. It was shown that surfactin interacts specifically with 1,2-dipalmitoylphosphatidylcholin (DPPC) localized at the DPPC/1,2-dioleoyl-sn-glycero-3-phosphocholine (DOPC) domain boundaries (Lins and Brasseur, 1995; Deleu et al., 2003, 2013).

For medical applications, the interaction of surfactin with the amyloid β -peptide (A β 42) has been studied with MD simulation and docking experiments [with GROMACS (Abraham et al., 2015) and AutoDock (Morris et al., 2009) respectively].

Further investigations have shown that surfactin binds protofibrils by forming a stable hydrogen bond with residues involved in salt bridges responsible ofamyloid aggregation and plaques stability (Verma et al., 2016). Another docking investigation, employing Swiss Dock (Lien Grosdidier et al., 2011), has shown that surfactin binds favorably via hydrogen bonds to porcine pancreatic lipase and inhibits its activity, which could lead to a novel and potent body weight reducer for obesity control (Meena et al., 2018).

Beside these investigations on monomeric surfactin interacting with potential targets, MD simulations proved to be an efficient tool to study molecular assemblies. A surfactin monolayer at the air-water interface was studied under various interfacial concentrations. It was shown that packed structures are formed via intra- and inter-molecular hydrogen bonds, stabilizing the β-turn structure of the peptide ring, favoring the β-sheet domain organization and hydrophobic contacts between molecules Another simulation was applied to study the self-assembly of surfactin in water and more particularly the structural organization of the micelles (Lebecque et al., 2017). Micelles were pre-formed with PackMol (Martinez et al., 2009) and were simulated to analyse their behavior. The optimal aggregation number, i.e., 20, predicted by this approach is in good agreement with the experimental values. Two parameters were analyzed, the hydrophilic (phi)/hydrophobic (pho) surface and the hydrophobic tail hydration (Lebecque et al., 2017). A higher phi/pho surface ratio means a more thermodynamically favorable organization of the hydrophilic and hydrophobic domains, but steric and/or electrical repulsions between polar heads have also to be considered. For surfactin, it was shown that the phi/pho surface ratio undergoes a decrease for the largest micelles of surfactin because they have to rearrange themselves to reach a more favorable organization. The low value of apolar moieties hydration observed for surfactin micelles is due to the very large peptidic head that efficiently preserves hydrophobic tails from contact with water. The Coarse Grain (CG) representation MARTINI (Marrink et al., 2007) (grouping atoms into beads to speed up the simulation process) was similarly applied to analyse the structural properties and kinetics of surfactin self-assembly in aqueous solution and at octane/water interface (Gang et al., 2020). With complementary MD of a pre-formed micelle and a monolayer, the authors showed that their CG model is in agreement with atomistic MD and experimental data, for micelle self-assembly and stability, as well as for the monolayer. Furthermore, this study allows the development of a set of optimized parameters in a MARTINI CG model that could open further investigations for surfactin interaction with various biofilms, proteins or other targets of interest with a better sampling than atomistic MD.

## Production

This last part of this review is dedicated to the improvement of the production of surfactin like compounds. It will first consider the techniques for the identification and the quantification of these lipopeptides and then focus on strain, culture conditions, and bioprocess optimization. Not to forget, the purification process allows for a greater recovery of the surfactin produced and lower the losses.

### Identification and Quantification of Surfactin and Its Variants

In order to discover new natural variants or verify the production of synthetic ones, the identification is an important process. The first surfactin structure elucidation was made through hydrolysis of the peptide and fatty acid chain into fragments, their identification and alignment (Kakinuma et al., 1969b). However, with the continuous innovations of analytical-chemical techniques such as mass spectrometry MS/MS (Yang et al., 2015a), nuclear magnetic resonance (NMR) (Kowall et al., 1998) and Fourier transform IR spectroscopy (FT-IR) (Fenibo et al., 2019), the analysis of new variants can be determined quicker and without hydrolysis. While FT-IR provides the functional groups, NMR leads to a complete structural characterization of the compounds but requires completely purified products at the level of mg quantities. Mass spectrometry does not enable the differentiation of compounds having the same mass (such as leucine and isoleucine for example), nor the type of fatty acid chain (linear, iso or anteiso), but provides the global mass and the peptide moiety primary sequence.

An overview of surfactin's dosage techniques can be found in Table 1. The first ones rely on surfactin's amphiphilic nature, so that its production can be detected through its surfactant activity.

**Table 1:** Techniques for detection and/or quantification of lipopeptide production.

**Technique**	**Advantages**	**Disadvantages**
Blood agar lysis	Ease of use	Not specific and not reliable
Drop collapse	Ease of use	Not specific
Oil spreading	Ease of use, better prediction than drop collapse	Not specific
Surface tension measurement	Ease of use, reliable	Not specific
Color shift	Ease of use, high-throughput	Not specific
HPLC-UV	Can discriminates the different lipopeptides if standard, quantification possible	Expensive equipment
LC-MS	Discriminates the different lipopeptides	Expensive equipment
PCR or genome sequencing	Production capacity measurement	Observes only genes
RT-PCR	Production capacity measurement	Observes only gene transcription

Indirect methods, such as emulsification measure, haemolytic activity (blood agar plate) or cell surface hydrophobicity can be used. However, the correlation between those activities and surfactant activity has been refuted. Youssef et al. (2004) does not recommend the use of blood agar lysis as a screening method. Therefore, direct methods to measure the surface activity, such as interfacial tension measurement, drop shape analysis, drop collapse assay or oil spreading should be used (Youssef et al., 2004). Newer techniques have been developed the last few years for a rapid detection and quantification, based on color shifts or fluorescence.

The first color shift approach is based on the higher affinity of a mediator, initially forming a complex with a color indicator, for surfactin and thus the release of the color indicator in the solution (Yang et al., 2015b). The fluorescence technique is based on the same principle, but with fluorescein instead of a color indicator (Heuson et al., 2018). This leads to a more sensitive and stable procedure. However, another color shift approach has been developed based only on the interaction between bromothymol blue solution and lipopeptides (Ong and Wu, 2018). However, since they are not specific for surfactin, the best and most sensitive quantification method is still the use of reversed phase HPLC-UV or MS (Geissler et al., 2017). This method also allows the discrimination between the various homologs of the surfactin family. Indeed, the molecules are separated based on their hydrophobic properties, giving a shorter retention time for lipopeptides with a leucine in position 7 and a longer retention time for lipopeptides with a valine in position 7. The separation is also based on the fatty acid chain, the shorter the fatty acid chain length is, the shorter the elution time is (Dhali, 2016). Furthermore, the production capacity of a micro-organism can be discovered through PCR, with primers specific to the surfactin biosynthesis genes (*sfp* and *srf* ) (Mohammadipour et al., 2009) or genome sequencing. However, these methods do not reflect the real lipopeptide production, since only the presence of the genes is observed. RT-PCR allows the detection of the transcribed genes, but does not allow to reflect the post-transcriptional modifications.

### Optimisation of Surfactin Production

In order to enhance the surfactin production, in addition to fermentation optimization, the genetic engineering of the producing strains is of great significance. It was already covered in the past by other teams (Hu et al., 2019) and will be more developed here.

**Figure 5:**
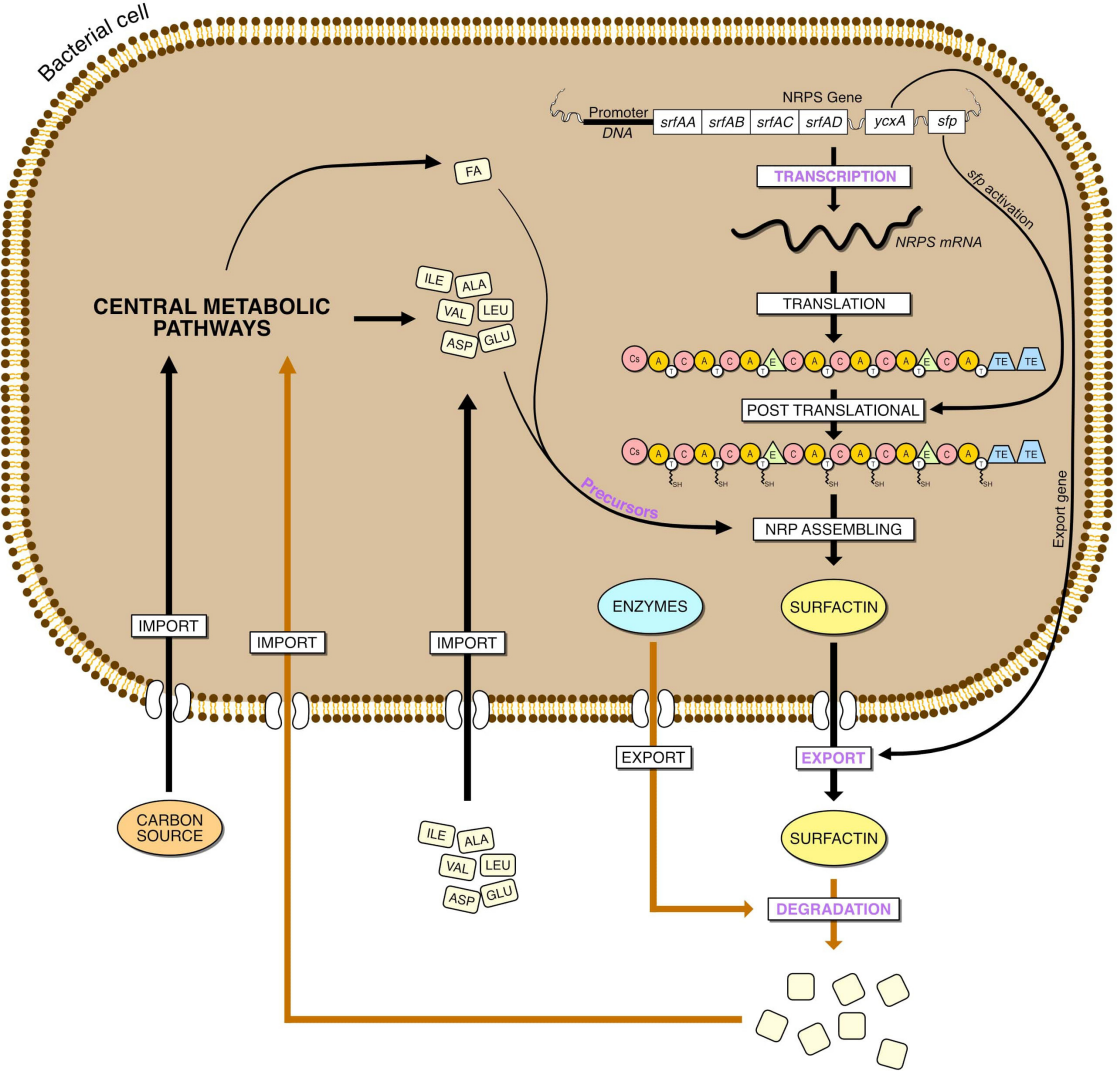
Steps involved in the overproduction of surfactin in Bacillus, from the gene expression to the degradation. The main steps are in purple, the yellow arrow represent hypothetical reactions.

A first strategy would be to allocate more resources of the cell to surfactin biosynthesis by suppressing different cellular processes. It was successful with the plipastatin operon disruption (Coutte et al., 2010a) or biofilm formation related genes (Wu et al., 2019). However, a strain with a 10 % genome deletion, comprising genes for plipastin, bacilysin, toxins, prophages and sporulation, had a lower surfactin production (Geissler et al., 2019). Then, concerning surfactin production itself, the strategy can take place at different stages of the surfactin cell production: at the transcription level by promoter substitution or modification of the transcriptional regulatory genes of *srfA* operon, at the level of surfactin synthesis by increasing the precursor availability, during the molecule's excretion and finally during its degradation (Figure 5).

#### Transcription

As seen before, surfactin NRPS is coded by four genes, *srfA-A, srfA-B, srfA-C*, and *srfA-D*, that are controlled by the *P*_*srf*_ antoinducible promoter, triggered by signal molecules from a quorum sensing pathway. Studies were performed to exchange this promoter with inducer-specific or constitutive ones. It emerged that a replacement with a constitutive promoter in a weak surfactin producer strain leads to an increase in the production, but that the opposite effect is observed for strong surfactin producers (Willenbacher et al., 2016). However, the use of novel artificial inducible promoters leads to an increase in surfactin production of more than 17 times (Jiao et al., 2017).

In addition to the promoter, transcriptional regulatory genes also control the expression of the NRPS genes. The cell density dependent quorum sensing system plays a regulatory role in many pathways in *Bacillus*, and among others in the regulation of the *srfA* operon. Ohsawa et al. (2006) showed that the inhibition of the ComQXP quorum sensing locus lead to a decrease in the expression of *srfA* genes and Jung et al. (2012) showed that the overexpression of ComX and PhrC increases the production of surfactin.

In addition to the quorum sensing system itself, regulators also impact the *srfA* operon, the quorum sensing system or even other mechanisms that indirectly impact surfactin. There are positive regulators such as PerR (Hayashi et al., 2005) and negative regulators such as CodY (Coutte et al., 2015), Rap (Hayashi et al., 2006), SinI (López et al., 2009) and Spx (Zhang et al., 2006).

#### Increasing Precursor Supply of NRPS by Feeding or Metabolic Engineering

Modifying media and fermentation condition is a strategy to overproduce the lipopeptide precursors as well as to favor the production of certain isoforms. For example it was seen that the feeding of leucine as 50% of the nitrogen source lead to an increase in specific surfactin production of three times (Coutte et al., 2015). Another strategy is the application of rational metabolic engineering approaches such as: (i) blocking competitive pathways for building blocks, as well as, those pathways that consume products; (ii) pulling flux through biosynthetic pathways by removing regulatory signals; and (iii) by overexpressing rate-limiting enzymes.

##### Amino Acids Precursors

One way to develop this metabolic engineering approach is to use knockout of genes which negatively influence the intracellular pool of amino acids precursors. To implement the knock-out of gene which negatively influence the intracellular pool of amino acid precursor, their metabolic pathways have to be modeled as a reaction network taking into account the regulation processes.

Firstly, the various pathways involved in the metabolites needed for the amino acid production should be addressed. In this research for compounds from the glycolysis that influence the amino acid production, pyruvate is interesting from multiple points of view. It is the entry point of the Krebs cycle through its conversion into acetyl-CoA, but it is also used as a substrate for the production of amino acids that compose the surfactin. Indeed, pyruvate is converted into valine and leucine. Furthermore, the production of isoleucine is made through threonine and pyruvate. The Krebs cycle also contributes to the amino acid production, with oxoglutarate and oxaloacetate, they belong to the metabolism of aspartic and glutamic acid. Secondly, the various enzymes that regulates metabolite production should be addressed. The search can also go a level above, with the regulators and promoters of those enzymes, such as pleiotropic regulators CodY or TnrA (Dhali, 2016). Lastly, the transporters of the amino acid precursors can be addressed. Indeed, the amino acid can be transported into the cell from the environment.

Wang et al. (2019), showed that the knockout of *murC, yrpC* and *racE*, negative regulators involved in the metabolism of glutamate, lead to an increase in surfactin production. The choice of those knock-outs can also be directed by methods from computational biology, to narrow them down and reduce the laboratory time needed.

Some prediction methods are based on formal reasoning techniques based on abstract-interpretation (Niehren et al., 2016). This is a general framework for abstracting formal models that is widely used in the static analysis of programming languages. Formal models are reaction networks with partial kinetic information with steady state semantics define systems of linear equations, with kinetic constraints, that are then abstracted. Here, the methods were to be developed further, so that they could be applied to reaction networks rather than other kinds of programs. This approach has been used for the branched chain amino acids (leucine, valine, and isoleucine) that mainly compose the surfactin peptide chain (Coutte et al., 2015).

The quite complex metabolic pathway of leucine production from threonine and pyruvate was modeled, by rewriting the informal model from SubtiWiki (Coutte et al., 2015) into this formal modeling language, while adding and adapting some reactions. It selected gene knock-outs that may lead to leucine overproduction, for which some of them an increase in surfactin production in *Bacillus subtilis* 168 was observed after experimental verification (Dhali et al., 2017).

Since single gene deletion is successful, multiple gene deletion must be the next aim. To be able to perform various deletions and/or insertions in the same strain, a markerless strategy is required. Various strategies can be performed such as temperature sensitive plasmid, pORI vectors, auxotrophy based methods, but also the *cre/lox* system (Yan et al., 2008), the pop-in pop-out technique (Tanaka et al., 2013) and the CRISPRi technology (Wang et al., 2019).

##### Fatty Acid Precursors

As mentioned, fatty acids are one of the crucial components of surfactin, and modifications of this part of the molecule, such as length and isomerism, demonstrated to impact on the physicochemical properties and on the biological activity of lipopeptides (Dufour et al., 2005; De Faria et al., 2011; Henry et al., 2011; Liu et al., 2015; Dhali et al., 2017). Different metabolic engineering strategies were applied to improve surfactin production, in terms of the branched-chain fatty acid supply included: (i) enhancing the branched-chain α-ketoacyl-CoA supply (Dhali et al., 2017; Wang et al., 2019; Wu et al., 2019); (ii) enhancing malonyl-ACP synthesis (Wu et al., 2019); (iii) overexpressing the whole fatty acid synthase complex (Wu et al., 2019); and (iv) pulling substrates flux toward surfactin biosynthesis by enhancing *srfA* transcription (Jiao et al., 2017; Wu et al., 2019).

Another study showed that the overexpression of the *bkd* operon produces less surfactin, besides being detrimental for cell growth (Wu et al., 2019). As the BKD complex requires lipoylation for its dehydrogenase activity, this enzyme competes with other lipoic acid dependent complexes (pyruvate dehydrogenase complex (PDH), 2-oxoacid dehydrogenase, acetoin dehydrogenase and the glycine cleavage system), generating a suppression of cell growth and, eventually, of surfactin production. By overexpressing the enzymes responsible for lipoic acid synthesis (*lipA, lipL*, and *lipM*) (Christensen et al., 2011; Martin et al., 2011), this suppressive effect is reversed. The competitive lipoylation process between BKD and other lipoic acid dependent complexes is eliminated (Wu et al., 2019) and thus generates a higher production of surfactin with respect to the parental strain.

A further pathway, targeted to modification, represents the malonyl-ACP synthesis. Acetyl-CoA is converted into malonyl-CoA through the activity of ACC (*accDABC*). Thus, overexpression of these genes in combination with that of *fabD*, the malonyl-CoA:ACP transacylase, has been reported to increase the levels of surfactin production (Wu et al., 2019). Furthermore, these authors applied systematic metabolic engineering in *B. subtilis* 168 to construct surfactin hyperproducer strains. Other successful interventions related to FA biosynthesis have also been described. The simultaneous overexpression of most FAS II coding genes; *fabH* and *fabGZIF* (Runguphan and Keasling, 2014) and expression of the *E. coli tesA* thioesterase (Steen et al., 2010), to “pull” through the pathway. The combination of the mentioned interventions, in an already modified *B. subtilis* 168 chassis, further improved surfactin production by 220% (Wu et al., 2019).

Acetyl-CoA, is a key intermediate metabolite, which is not only used for surfactin biosynthesis, but fundamentally for cell growth and proliferation. Acetyl-CoA is generated from pyruvate by PDH; overexpression of enzymes of the glycolytic pathway and the KO of genes coding for enzymes associated with the acetyl-CoA consumption are common strategies to increase the supply of this key intermediate. Wu et al. (2019) showed that the simultaneous overexpression of the PDH genes and that of the glycolysis enzymes produce an increase in biomass but not a significant increase in the levels of surfactin. However, if these interventions were combined with the overexpression/deregulation of the *srf* gene cluster, the surfactin production could be further improved to 12.8 g/l, achieving a 42% (mmol surfactin/mol sucrose) of the theoretical yield.

##### Directed Biosynthesis of Surfactin

D Due to the non-specificity of some adenylation domains, the proportion of natural variants of surfactin can be modified through the feeding of certain amino acids as the nitrogen source in the culture medium. In the peptide moiety, this only affects L amino acid residues located in position 2, 4, and 7, and with a greater variation in position 4. Indeed, the feeding of valine leads to an increase of valine in position 7 (Menkhaus et al., 1993), the feeding of isoleucine (Ile) leads to the apparition of isoleucine in position 2 and/or 4 (Grangemard et al., 1997) and the feeding of alanine (Ala) lead to a surfactin with alanine in position 4 (Peypoux et al., 1994). Also, the culture medium can also influence the proportion of surfactin variants with different acyl moieties. For example, Liu et al. (Liu et al., 2015) found that the strain *B. subtilis* BS-37 has lower surfactin titers with higher proportions of C15-surfactin when grown in LB compared with glucose medium. Another team analyzed the influence of amino acid residues on the pattern of surfactin variants produced by *B. subtilis* TD7 (Liu et al., 2012). The β-hydroxy fatty acid in surfactin variants was C15>C14>C13>C16, when no amino acid was added in the culture medium. On the other hand, when Arg, Gln, or Val was added to the culture medium, the proportion of surfactins with even β-hydroxy fatty acid chain significantly increased; whereas the addition of Cys, His, Ile, Leu, Met, Ser, or Thr significantly enhanced the proportion of surfactins with odd β-hydroxy fatty acid. Some of these results can be explained by the mode of biosynthesis of branched fatty acids, the precursors of which are branched chain amino acids (Kaneda, 1991). Thus, valine feeding enhances the proportion of iso variants with even fatty acid chains, while leucine and isoleucine feeding enhances the proportion of uneven iso or anteiso fatty acids chains respectively (Liu et al., 2012).

Modification of the variant pattern can also be obtained by genetic engineering of precursor pathways. As previously mentioned, increasing the branched chain 2-ketoacyl-CoAs intermediates is one of the strategies used for enhancing the synthesis of surfactin. The deletion of gene *codY*, which encodes a global transcriptional regulator and negatively regulates the *bkd* operon lead to a 5.8-fold increase in surfactin production in *B. subtilis* BBG258 with an increase by a factor 1.4 of the amino acid valine in position 7 instead of leucine (Dhali et al., 2017). On the other hand, Wang et al. (2019), using CRISPR interference (CRISPRi) technology, were able to repress the *bkdAA* and *bkdAB* genes of the *bkd* operon; provoking a modest improvement in surfactin concentration, but a significant change in the proportion of the nC14 component. Similar results were observed in *B. subtilis* BBG261, a derivative lpdV mutant strain, where the interruption of this 2-oxoisovalerate dehydrogenase of the BKD complex led to higher percentage of the nC14 isoform (52,7% in the *lpdV* mutant in comparison with the 21,2% of the control strain) (Dhali et al., 2017).

#### Excretion

The excretion of surfactin is another important step for its overproduction. Even if, as mentioned before, surfactin can insert itself in the membrane of the cell, the transmembrane efflux is mediated by protein transporters.

As mentioned before, thanks to its amphiphilic structure, surfactin can interact with the membrane of the cell. Under or at the CMC, the surfactin can insert itself in the membrane, and above the CMC it can even solubilize it (Deleu et al., 2003, 2013). However, it was hypothesized by Tsuge et al. that the gene y*erP*, homolog to the RND family efflux pumps, is involved in the surfactin efflux (Tsuge et al., 2001). Later, Li et al. (2015) showed that the overexpression of three lipopeptide transporters, dependent on proton motive force, YcxA, KrsE and YerP lead to an increase in surfactin export of 89, 52, and 145% respectively.

Those studies are promising and the efflux proteins need to be further investigated to fully understand the excretion of surfactin.

#### Degradation

Lastly, the importance of surfactin degradation should not be underestimated. Indeed, a decrease in surfactin concentration of 59 and 73% has been observed during the fermentation process (Nitschke and Pastore, 2004; Maass et al., 2016), leading to the presence of degradation mechanisms by the cell themselves.

Three hypotheses are considered by the different teams observing this phenomenon. Since that, for different mediums with the same carbon content, the surfactin decrease happened at the same time, it could be that surfactin is used as a carbon source after glucose depletion. Or, since the decrease happened at the same surfactin concentration, that it is degraded because of its possible inhibitory effect at higher concentration (Maass et al., 2016). It was also shown that the surfactin decrease is linked to the increase in protease activity in the culture medium and thus the produced enzymes could be involved in this degradation (Nitschke and Pastore, 2004).

As for the excretion, this degradation process was seldomly researched but could greatly influence the surfactin production.

### Culture Medium and Conditions

Landy culture medium, based on glucose and glutamic acid, is one the main culture medium usually used for surfactin production. Furthermore, some studies have been performed to ameliorate it (Jacques et al., 1999; Akpa et al., 2001; Wei et al., 2007; Ghribi and Ellouze-Chaabouni, 2011; Huang et al., 2015; Willenbacher et al., 2015).

However, another type of approach for the culture medium is rising. Indeed, the use of cheap substrate such as waste or by-products from the agro-industrial field is more and more researched (De Faria et al., 2011; Gudiña et al., 2015; Moya Ramírez et al., 2015; Paraszkiewicz et al., 2018), since this approach enables a sustainable production of surfactins. The recent review of Zanotto et al. develops specifically this approach (Zanotto et al., 2019).

Concerning the fundamental parameters of culture condition, a pH of 7 and a temperature of 37°C leads to a higher production rate (Ohno et al., 1995a). However, when up-scaling from a flask culture to a larger scale, the main challenge in surfactin production appears. Indeed, the agitation rate and oxygenation of the culture medium play an important role in the production (Hbid et al., 1996; Guez et al., 2008; Ghribi and Ellouze-Chaabouni, 2011). As surfactin is a surfactant and thus increases the stability of a gas-liquid dispersion, this agitation leads to the abundant production of foam. Nonetheless, even if this foam production is often considered as a drawback, it can be used with the appropriate reactors as an advantage to easily recover surfactin.

### Production Processes

For an overproduction of surfactin, the addition of a solid carrier to an agitated liquid culture can enhance surfactin production by stimulating cell growth and by promoting a biofilm formation. Yeh et al. (2005) added activated carbon, agar and expanded clay, observing a 36 times increase with activated carbon.

Nonetheless, as mentioned before, due to the high foam generation in surfactin production, classical stirred reactors are not optimal for this bioprocess. Indeed, adding antifoam to the culture medium has many drawbacks. Antifoams may have a negative effect on cell growth and are costly, but even more, they have to be eliminated during purification. Thus, multiple strategies can be applied: (i) to use this foam production to its advantage or (ii) to reduce or avoid foam production.

For the first strategy, the foam fractionation method consists in a continuous removal of the foam from a liquid agitated culture to a sterile vessel. So, this removal is a first purification step and by the continuous extraction avoids any possible feedback inhibition from the products (Cooper et al., 1981; Davis et al., 2001). However, the foam can carry a part of the culture medium and cells out and thus decrease the production. For the second strategy, a rotating disk bioreactor was used by Chtioui et al. (2012) where a biofilm formation occurs on a rotating disk in a liquid medium. The process is simple and can easily be upscaled, but the oxygen transfer is quite low and thus not optimal for surfactin production.

*Bacillus* biofilm formation capacity can also be used in other type of biofilm reactors such as packed bed reactors, where the liquid medium recirculates on a packing in the reactor (Zune et al., 2016). The purification is easily performed, but the biofilm growth is difficult to control because it depends on the liquid distribution in the packing. Recent studies have considered the genetic engineering of the bacterial cells to modify their biofilm formation ability or their filamentous growth in order to enhance their adhesion on the packing (Brück et al., 2019, 2020).

A membrane reactor allows for a bubbleless oxygen transfer through a membrane between the air and the culture medium. Furthermore, a first surfactin purification can be made through ultrafiltration coupled to the fermentation (Coutte et al., 2010b). However, there is a surfactin adsorption on the membrane and they can be costly when upscaled.

Lastly, a solid medium can be used with solid state fermentation that avoids the mechanical stirring of liquid cultures and thus the foam production. It represents a simple process but with parameters more difficult to control than in a liquid culture. However, many waste and by-products used as novel substrate are in a solid state and could thus be used without pretreatment (Ohno et al., 1995b).

Most studies are performed on the enhancement of one of the steps of the production process, but some studies are performed to decrease the costs in a large scale production (Czinkóczky and Németh, 2020).

### Purification

The purification process is a major step in the surfactin production and depends on the fermentation process used. Linked to the techniques mentioned before, foam can be recovered during the fermentation and lead to 70% of recovery (Davis et al., 2001; Willenbacher et al., 2014). For a fermentation process with the surfactin in the liquid medium, acid precipitation, linked to the negative charge of surfactin, is the oldest and more common used technique. It can lead to a high recovery rate, but has a low purity (55%) and is the only technique that cannot be continuously coupled to the production. Solvent extraction can also be used alone but it is mostly coupled with acid precipitation to enhance the purity (Kim et al., 1997; Geissler et al., 2017). One of the most common type of purification, membrane filtration, can especially be used for surfactin through its micelle forming ability above its critical micelle concentration. The aggregated molecule is larger an thus can be retained by membranes with a MWCO of 10–100 kDa (Jauregi et al., 2013) with recovery rates and a purity above 90% depending on the applied membrane. Furthermore, hybrid methods have been successfully employed, i.e., precipitation before filtration (Chen et al., 2007), which facilitated the process or increased the final purity.

The techniques mentioned above are mostly used for the extraction of surfactin from the culture medium. Some uses of surfactin require a higher purity that can be obtained with the following methods. The physico-chemical properties of surfactin can be used through its adsorption on resin or active charcoal (Liu et al., 2007), leading to variable recovery rates and purity. Chromatographic derived methods can also be used to get a better purity and to separate individual variants or isoforms of the lipopeptide (Smyth et al., 2010). Reverse phase chromatography, based on hydrophobic interactions, is the most common technique employed.

## Conclusions

With the improved genetic toolbox which is now available, a larger and more diverse chemical space of the surfactin scaffold can be generated and explored. This endeavor will create novel surfactin derivatives with improved, specialized, or expanded biological activities. And even if this molecule's potential applications range is already broad and reaches different industrials sectors, it may be enhanced with those novel compounds. However, despite the advancements in surfactin production, its production cost is still withholding it for a widespread commercial use in low added-value applications.

## Data Availability Statement

The original contributions presented in the study are included in the article/supplementary material, further inquiries can be directed to the corresponding authors.

## Author Contributions

The literature review and manuscript writing were performed by AT, CC-P, MB, YL, MD, JN, TF, SG, AL, LL, AA, HGra, HGro, and PJ. Insights were provided by MA and MM. In addition, AT and PJ have co-ordinated and synthesized the different contributions. All authors have read and agreed to the published version of the review.

## Conflict of Interest

PJ is a co-founder of Lipofabrik and Lipofabrik Belgium and a member of the scientific advisory board of both companies. MM is a co-founder of Design Pharmaceuticals and a member of the scientific advisory board of Hexagon Bio. The remaining authors declare that the research was conducted in the absence of any commercial or financial relationships that could be construed as a potential conflict of interest.

## References

[B1] Abraham M. J. Murtola T. Schulz R. Pall S. Smith J. C. Hess B.. (2015). Gromacs: high performance molecular simulations through multi-level parallelism from laptops to supercomputers. SoftwareX 1–2, 19–25. 10.1016/j.softx.2015.06.001

[B2] Ahimou F. Jacques P. Deleu M. (2000). Surfactin and iturin A effects on *Bacillus subtilis* surface hydrophobicity. Enzyme Microb. Technol. 27, 749–754. 10.1016/S0141-0229(00)00295-7 11118581

[B3] Akpa E. Jacques P. Wathelet B. Paquot M. Fuchs R. Budzikiewicz H.. (2001). Influence of culture conditions on lipopeptide production by *Bacillus subtilis*. Appl. Biochem. Biotechnol. 91–93, 551–561. 10.1385/ABAB:91-93:1-9:551 11963884

[B4] Alanjary M. Cano-Prieto C. Gross H. Medema M. H. (2019). Computer-aided re-engineering of nonribosomal peptide and polyketide biosynthetic assembly lines. Nat. Prod. Rep. 36, 1249–1261. 10.1039/C9NP00021F 31259995

[B5] Alvarez V. M. Guimarães C. R. Jurelevicius D. de Castilho L. V. A. de Sousa J. S. da Mota F. F.. (2020). Microbial enhanced oil recovery potential of surfactin-producing *Bacillus subtilis* AB2.0. Fuel 272:117730. 10.1016/j.fuel.2020.117730

[B6] Ansaldi M. Marolt D. Stebe T. Mandic-Mulec I. Dubnau D. (2002). Specific activation of the *Bacillus* quorum-sensing systems by isoprenylated pheromone variants. Mol. Microbiol. 44, 1561–1573. 10.1046/j.1365-2958.2002.02977.x 12067344

[B7] Auchtung J. M. Lee C. A. Grossman A. D. (2006). Modulation of the ComA-dependent quorum response in *Bacillus subtilis* by multiple rap proteins and Phr peptides. J. Bacteriol. 188, 5273–5285. 10.1128/JB.00300-06 16816200 PMC1539962

[B8] Bernheimer A. W. Avigad L. S. (1970). Nature and properties of a cytolytic agent produced by Bacillus subtilis. J. Gen. Microbiol. 61, 361–369. 10.1099/00221287-61-3-361 4992273

[B9] Bloudoff K. Schmeing T. M. (2017). Structural and functional aspects of the nonribosomal peptide synthetase condensation domain superfamily: discovery, dissection and diversity. Biochim. Biophys. Acta Proteins Proteom. (1865) 1587–1604. 10.1016/j.bbapap.2017.05.010 28526268

[B10] Bonmatin J.-M. Laprevote O. Peypoux F. (2003). Diversity among microbial cyclic lipopeptides: iturins and surfactins. Activity-structure relationships to design new bioactive agents. Comb. Chem. High Throughput Screen. 6, 541–556. 10.2174/138620703106298716 14529379

[B11] Bozhüyük K. A. J. Linck A. Tietze A. Kranz J. Wesche F. Nowak S.. (2019). Modification and *de novo* design of non-ribosomal peptide synthetases using specific assembly points within condensation domains. Nat. Chem. 11, 653–661. 10.1038/s41557-019-0276-z 31182822

[B12] Brasseur R. Killian J. A. De Kruijff B. Ruysschaert J. M. (1987). Conformational analysis of gramicidin-gramicidin interactions at the air/water interface suggests that gramicidin aggregates into tube-like structures similar as found in the gramicidin-induced hexagonal HII phase. BBA - Biomembr. 903, 11–17. 10.1016/0005-2736(87)90150-7 2443166

[B13] Brück H. L. Coutte F. Dhulster P. Gofflot S. Jacques P. Delvigne F. (2020). Growth dynamics of bacterial populations in a two-compartment biofilm bioreactor designed for continuous surfactin biosynthesis. Microorganisms 8:679. 10.3390/microorganisms8050679 32392736 PMC7285194

[B14] Brück H. L. Delvigne F. Dhulster P. Jacques P. Coutte F. (2019). Molecular strategies for adapting Bacillus subtilis 168 biosurfactant production to biofilm cultivation mode. Bioresour. Technol. 293:122090. 10.1016/j.biortech.2019.122090 31499329

[B15] Chandler S. Van Hese N. Coutte F. Jacques P. Höfte M. De Vleesschauwer D. (2015). Role of cyclic lipopeptides produced by *Bacillus subtilis* in mounting induced immunity in rice (*Oryza sativa* L.). Physiol. Mol. Plant Pathol. 91, 20–30. 10.1016/j.pmpp.2015.05.010

[B16] Chen H. L. Chen Y. S. Juang R. S. (2007). Separation of surfactin from fermentation broths by acid precipitation and two-stage dead-end ultrafiltration processes. J. Memb. Sci. 299, 114–121. 10.1016/j.memsci.2007.04.031

[B17] Choi K. H. Heath R. J. Rock C. O. (2000). β-ketoacyl-acyl carrier protein synthase III (FabH) is a determining factor in branched-chain fatty acid biosynthesis. J. Bacteriol. 182, 365–370. 10.1128/JB.182.2.365-370.2000 10629181 PMC94284

[B18] Christensen Q. H. Martin N. Mansilla M. C. de Mendoza D. Cronan J. E. (2011). A novel amidotransferase required for lipoic acid cofactor assembly in *Bacillus subtilis*. Mol Microbiol. 80, 350–363. 10.1111/j.1365-2958.2011.07598.x 21338421 PMC3088481

[B19] Chtioui O. Dimitrov K. Gancel F. Dhulster P. Nikov I. (2012). Rotating discs bioreactor, a new tool for lipopeptides production. Process Biochem. 47, 2020–2024. 10.1016/j.procbio.2012.07.013

[B20] Cooper D. G. Macdonald C. R. Duff S. J. B. B. Kosaric N. (1981). Enhanced production of surfactin from bacillus subtilis by continuous product removal and metal cation additions. Appl. Environ. Microbiol. 42, 408–412. 10.1128/AEM.42.3.408-412.1981 16345840 PMC244028

[B21] Cosmina P. Rodriguez F. de Ferra F. Grandi G. Perego M. Venema G.. (1993). Sequence and analysis of the genetic locus responsible for surfactin synthesis in *Bacillus subtilis*. Mol. Microbiol. 8, 821–831. 10.1111/j.1365-2958.1993.tb01629.x 8355609

[B22] Coutte F. Leclère V. Béchet M. Guez J. S. Lecouturier D. Chollet-Imbert M.. (2010a). Effect of pps disruption and constitutive expression of srfA on surfactin productivity, spreading and antagonistic properties of Bacillus subtilis 168 derivatives. J. Appl. Microbiol. 109, 480–491. 10.1111/j.1365-2672.2010.04683.x 20148996

[B23] Coutte F. Lecouturier D. Yahia S. A. Leclère V. Béchet M. Jacques P.. (2010b). Production of surfactin and fengycin by Bacillus subtilis in a bubbleless membrane bioreactor. Appl. Microbiol. Biotechnol. 87, 499–507. 10.1007/s00253-010-2504-8 20221757

[B24] Coutte F. Niehren J. Dhali D. John M. Versari C. Jacques P. (2015). Modeling leucine's metabolic pathway and knockout prediction improving the production of surfactin, a biosurfactant from *Bacillus subtilis*. Biotechnol. J. 10, 1216–1234. 10.1002/biot.201400541 26220295

[B25] Czinkóczky R. Németh Á. (2020). Techno-economic assessment of Bacillus fermentation to produce surfactin and lichenysin. Biochem. Eng. J. 163:107719. 10.1016/j.bej.2020.107719

[B26] Davis D. A. Lynch H. C. Varley J. (2001). The application of foaming for the recovery of Surfactin from *B. subtilis* ATCC 21332 cultures. Enzyme Microb. Technol. 28, 346–354. 10.1016/S0141-0229(00)00327-6 11240190

[B27] de Araujo L. L. G. C. Sodré L. G. P. Brasil L. R. Domingos D. F. de Oliveira V. M. da Cruz G. F. (2019). Microbial enhanced oil recovery using a biosurfactant produced by *Bacillus safensis* isolated from mangrove microbiota - Part I biosurfactant characterization and oil displacement test. J. Pet. Sci. Eng. 180, 950–957. 10.1016/j.petrol.2019.06.031

[B28] De Faria A. F. Teodoro-Martinez D. S. De Oliveira Barbosa G. N. Gontijo Vaz B. Serrano Silva Í. Garcia J. S.. (2011). Production and structural characterization of surfactin (C 14/Leu7) produced by *Bacillus subtilis*isolate LSFM-05 grown on raw glycerol from the biodiesel industry. Process Biochem. 46, 1951–1957. 10.1016/j.procbio.2011.07.001

[B29] Deleu M. Bouffioux O. Razafindralambo H. Paquot M. Hbid C. Thonart P.. (2003). Interaction of surfactin with membranes: a computational approach. Langmuir 19, 3377–3385. 10.1021/la026543z

[B30] Deleu M. Lorent J. Lins L. Brasseur R. Braun N. El Kirat K.. (2013). Effects of surfactin on membrane models displaying lipid phase separation. Biochim. Biophys. Acta Biomembr. (1828). 801–815. 10.1016/j.bbamem.2012.11.007 23159483

[B31] Deleu M. Razafindralambo H. Popineau Y. Jacques P. Thonart P. Paquot M. (1999). Interfacial and emulsifying properties of lipopeptides from *Bacillus subtilis*. Colloids Surf. Physicochem. Eng. Asp. 152, 3–10. 10.1016/S0927-7757(98)00627-X18479055

[B32] Deravel J. Lemière S. Coutte F. Krier F. Van Hese N. Béchet M.. (2014). Mycosubtilin and surfactin are efficient, low ecotoxicity molecules for the biocontrol of lettuce downy mildew. Appl. Microbiol. Biotechnol. 98, 6255–6264. 10.1007/s00253-014-5663-1 24723290

[B33] Desmyttere H. Deweer C. Muchembled J. Sahmer K. Jacquin J. Coutte F.. (2019). Antifungal activities of bacillus subtilis lipopeptides to two venturia inaequalis strains possessing different tebuconazole sensitivity. Front. Microbiol. 10:2327. 10.3389/fmicb.2019.02327 31695685 PMC6817503

[B34] Dhali D. (2016). Correlation Between Lipopeptide Biosynthesis and their Precursor Metabolism in Bacillus subtilis.

[B35] Dhali D. Coutte F. Argüelles A. Auger S. Bidnenko V. Chataign,é G. . (2017). Genetic engineering of the branched fatty acid metabolic pathway of *Bacillus subtilis* for the overproduction of surfactin C14isoform. Biotechnol. J. 12, 1–23. 10.1002/biot.201600574 28371347

[B36] Dieckmann R. Lee Y. O. van Liempt H. von Döhren H. Kleinkauf H. (1995). Expression of an active adenylate-forming domain of peptide synthetases corresponding to acyl-CoA-synthetases. FEBS Lett. 357, 212–216. 10.1016/0014-5793(94)01342-X 7805893

[B37] D'Souza C. Nakano M. M. Zuber P. (1994). Identification of comS, a gene of the srfA operon that regulates the establishment of genetic competence in *Bacillus subtilis*. Proc. Natl. Acad. Sci. U.S.A. 91, 9397–9401. 10.1073/pnas.91.20.9397 7937777 PMC44819

[B38] Dufour S. Deleu M. Nott K. Wathelet B. Thonart P. Paquot M. (2005). Hemolytic activity of new linear surfactin analogs in relation to their physico-chemical properties. Biochim. Biophys. Acta - Gen. Subj. (1726). 87–95. 10.1016/j.bbagen.2005.06.015 16026933

[B39] Eeman M. Berquand A. Dufrêne Y. F. Paquot M. Dufour S. Deleu M. (2006). Penetration of surfactin into phospholipid monolayers: nanoscale interfacial organization. Langmuir 22, 11337–11345. 10.1021/la061969p 17154623

[B40] Eivazihollagh A. Svanedal I. Edlund H. Norgren M. (2019). On chelating surfactants: Molecular perspectives and application prospects. J. Mol. Liq. 278, 688–705. 10.1016/j.molliq.2019.01.076

[B41] Eppelmann K. Stachelhaus T. Marahiel M. A. (2002). Exploitation of the selectivity-conferring code of nonribosomal peptide synthetases for the rational design of novel peptide antibiotics. Biochemistry 41, 9718–9726. 10.1021/bi0259406 12135394

[B42] Fei D. Zhou G. Yu Z. Gang H. Liu J. Yang S.. (2020). Low-toxic and nonirritant biosurfactant surfactin and its performances in detergent formulations. J. Surfactants Deterg. 23, 109–118. 10.1002/jsde.12356

[B43] Fenibo E. O. Douglas S. I. Stanley H. O. (2019). A review on microbial surfactants: production, classifications, properties and characterization. J. Adv. Microbiol. 18, 1–22. 10.9734/jamb/2019/v18i330170

[B44] Francius G. Dufour S. Deleu M. Paquot M. Mingeot-Leclercq M. P. Dufrêne Y. F. (2008). Nanoscale membrane activity of surfactins: Influence of geometry, charge and hydrophobicity. Biochim. Biophys. Acta - Biomembr. 2058–2068. 10.1016/j.bbamem.2008.03.023 18455997

[B45] Fujita Y. Matsuoka H. Hirooka K. (2007). Regulation of fatty acid metabolism in bacteria. Mol. Microbiol. 66, 829–839. 10.1111/j.1365-2958.2007.05947.x 17919287

[B46] Fuma S. Fujishima Y. Corbell,' N Souzal C. D.', Nakano, M. M. Zuberl P. Yamane K. (1993). Nucleotide sequence of 5' portion of srfA that contains the region required for competence establishment in Bacillus subtilus. Nucleic Acids Res. 21, 93–97. 10.1093/nar/21.1.93 8441623 PMC309069

[B47] Gang H. He H. Yu Z. Wang Z. Liu J. He X.. (2020). A coarse-grained model for microbial lipopeptide surfactin and its application in self-assembly. J. Phys. Chem. (2020). 1839–1846. 10.1021/acs.jpcb.9b11381 32083878

[B48] Gao L. Han J. Liu H. Qu X. Lu Z. Bie X. (2017). Plipastatin and surfactin coproduction by *Bacillus subtilis* pB2-L and their effects on microorganisms. Antonie van Leeuwenhoek. Int. J. Gen. Mol. Microbiol. 110, 1007–1018. 10.1007/s10482-017-0874-y 28477175

[B49] Geissler M. Kühle I. Heravi K. M. Altenbuchner J. Henkel M. Hausmann R. (2019). Evaluation of surfactin synthesis in a genome reduced Bacillus subtilis strain. AMB Expr. 9:84. 10.1186/s13568-019-0806-5 31190306 PMC6562014

[B50] Geissler M. Oellig C. Moss K. Schwack W. Henkel M. Hausmann R. (2017). High-performance thin-layer chromatography (HPTLC) for the simultaneous quantification of the cyclic lipopeptides Surfactin, Iturin A and Fengycin in culture samples of *Bacillus species*. J. Chromatogr. B Anal. Technol. Biomed. Life Sci. 1044–1045, 214–224. 10.1016/j.jchromb.2016.11.013 28153674

[B51] Ghribi D. Ellouze-Chaabouni S. (2011). Enhancement of bacillus subtilis lipopeptide biosurfactants production through optimization of medium composition and adequate control of aeration. Biotechnol. Res. Int. (2011) 2011:653654. 10.4061/2011/653654 21966596 PMC3182341

[B52] Grangemard I. Peypoux F. Wallach J. Das B. C. Labb,é H Caille A.. (1997). Lipopeptides with improved properties: structure by NMR, purification by HPLC and structure-activity relationships of new isoleucyl-rich surfactins. J. Pept. Sci. 3, 145–154. 10.1002/(SICI)1099-1387(199703)3:2<145::AID-PSC96>3.0.CO;2-Y 9230480

[B53] Grangemard I. Wallach J. Maget-Dana R. Peypoux F. (2001). Lichenysin: A more efficient cation chelator than surfactin. Appl. Biochem. Biotechnol. 90, 199–210. 10.1385/ABAB:90:3:199 11318033

[B54] Grangemard I. Wallach J. Peypoux F. (1999). Evidence of surfactin hydrolysis by a bacterial endoprotease. Biotechnol. Lett. 21, 241–244. 10.1023/A:1005444717166

[B55] Graumann P. L. Marahiel M. A. (1999). Cold Shock Response in *Bacillus subtilis* JMMB Symposium. J. Mol. Microbiol. Biotechnol 1, 203–209. 10943551

[B56] Gudiña E. J. Fernandes E. C. Rodrigues A. I. Teixeira J. A. Rodrigues L. R. (2015). Biosurfactant production by Bacillus subtilis using corn steep liquor as culture medium. Front. Microbiol. 6:59. 10.3389/fmicb.2015.00059 25705209 PMC4319461

[B57] Guez J. S. Müller C. H. Danze P. M. Büchs J. Jacques P. (2008). Respiration activity monitoring system (RAMOS), an efficient tool to study the influence of the oxygen transfer rate on the synthesis of lipopeptide by Bacillus subtilis ATCC6633. J. Biotechnol. 134, 121–126. 10.1016/j.jbiotec.2008.01.003 18282625

[B58] Habe H. Taira T. Imura T. (2018). Surface activity and Ca 2+-dependent aggregation property of lichenysin produced by *Bacillus licheniformis* NBRC 104464. J. Oleo Sci. 67, 1307–1313. 10.5650/jos.ess18107 30305561

[B59] Hamoen L. W. Eshuis H. Jongbloed J. Venema G. van Sinderen D. (1995). A small gene, designated comS, located within the coding region of the fourth amino acid-activation domain of srfA, is required for competence development in *Bacillus subtilis*. Mol. Microbiol. 15, 55–63. 10.1111/j.1365-2958.1995.tb02220.x 7752896

[B60] Hayashi K. Kensuke T. Kobayashi K. Ogasawara N. Ogura M. (2006). *Bacillus subtilis* RghR (YvaN) represses *rapG* and *rapH*, which encode inhibitors of expression of the *srfA* operon. Mol. Microbiol. 59, 1714–1729. 10.1111/j.1365-2958.2006.05059.x 16553878

[B61] Hayashi K. Ohsawa T. Kobayashi K. Ogasawara N. Ogura M. (2005). The H2O2 stress-responsive regulator perr positively regulates *srfA* expression in *Bacillus subtilis*. J. Bacteriol. 187, 6659–6667. 10.1128/JB.187.19.6659-6667.2005 16166527 PMC1251593

[B62] Hbid C. Jacques P. Razafindralambo H. Mpoyo M. K. Meurice E. Paquot M.. (1996). Influence of the production of two lipopeptides, Iturin A and Surfactin S1, on oxygen transfer during *Bacillus subtilis* fermentation. Appl. Biochem. Biotechnol. 57–58, 571–579. 10.1007/BF02941737

[B63] Heath R. J. Su N. Murphy C. K. Rock C. O. (2000). The enoyl-[acyl-carrier-protein] reductases FabI and FabL from *Bacillus subtilis*. J. Biol. Chem. 275, 40128–40133. 10.1074/jbc.M005611200 11007778

[B64] Henry G. Deleu M. Jourdan E. Thonart P. Ongena M. (2011). The bacterial lipopeptide surfactin targets the lipid fraction of the plant plasma membrane to trigger immune-related defence responses. Cell. Microbiol. 13, 1824–1837. 10.1111/j.1462-5822.2011.01664.x 21838773

[B65] Heuson E. Etchegaray A. Filipe S. L. Beretta D. Chevalier M. Phalip V.. (2018). Screening of lipopeptide producing strains of Bacillus sp. using a new automated and sensitive fluorescence detection method. Biotechnol. J. 14:1800314. 10.1002/biot.201800314 30430761

[B66] Hlavica P. Lehnerer M. (2010). Oxidative biotransformation of fatty acids by cytochromes P450: predicted key structural elements orchestrating substrate specificity, regioselectivity and catalytic efficiency. Curr. Drug Metab. 11, 85–104. 10.2174/138920010791110881 20302567

[B67] Hoefler B. C. Gorzelnik K. V. Yang J. Y. Hendricks N. Dorrestein P. C. Straight P. D. (2012). Enzymatic resistance to the lipopeptide surfactin as identified through imaging mass spectrometry of bacterial competition. Proc. Natl. Acad. Sci. U.S.A. 109, 13082–13087. 10.1073/pnas.1205586109 22826229 PMC3420176

[B68] Horowitz S. Gilbert J. N. Griffin W. M. (1990). Isolation and characterization of a surfactant produced by *Bacillus licheniformis* 86. J. Ind. Microbiol. 6, 243–248. 10.1007/BF01575868

[B69] Hu F. Liu Y. Li S. (2019). Rational strain improvement for surfactin production: enhancing the yield and generating novel structures. Microb. Cell Fact. 18:42. 10.1186/s12934-019-1089-x 30819187 PMC6394072

[B70] Huang X. Liu J. Wang Y. Liu J. Lu L. (2015). The positive effects of Mn 2+ on nitrogen use and surfactin production by *Bacillus subtilis* ATCC 21332. Biotechnol. Biotechnol. Equip. 29, 381–389. 10.1080/13102818.2015.1006905 26019656 PMC4433937

[B71] Hwang Y. H. Park B. K. Lim J. H. Kim M. S. Park S. C. Hwang M. H.. (2007). Lipopolysaccharide-binding and neutralizing activities of surfactin C in experimental models of septic shock. Eur. J. Pharmacol. 556, 166–171. 10.1016/j.ejphar.2006.10.031 17126323

[B72] Jacques P. Hbid C. Destain J. Razafindralambo H. Paquot M. De Pauw E.. (1999). Optimization of biosurfactant lipopeptide production from *Bacillus subtilis* S499 by plackett-burman design. Appl. Biochem. Biotechnol. 77, 223–234. 10.1385/ABAB:77:1-3:223

[B73] Jahan R. Bodratti A. M. Tsianou M. Alexandridis P. (2020). Biosurfactants, natural alternatives to synthetic surfactants: physicochemical properties and applications. Adv. Colloid Interface Sci. 275:102061. 10.1016/j.cis.2019.102061 31767119

[B74] Jauregi P. Coutte F. Catiau L. Lecouturier D. Jacques P. (2013). Micelle size characterization of lipopeptides produced by *B. subtilis* and their recovery by the two-step ultrafiltration process. Sep. Purif. Technol. 104, 175–182. 10.1016/j.seppur.2012.11.017

[B75] Jiang J. Gao L. Bie X. Lu Z. Liu H. Zhang C.. (2016). Identification of novel surfactin derivatives from NRPS modification of *Bacillus subtilis* and its antifungal activity against *Fusarium moniliforme*. BMC Microbiol. 16:31. 10.1186/s12866-016-0645-3 26957318 PMC4784341

[B76] Jiao S. Li X. Yu H. Yang H. Li X. Shen Z. (2017). In situ enhancement of surfactin biosynthesis in *Bacillus subtilis* using novel artificial inducible promoters. Biotechnol. Bioeng. 114, 832–842. 10.1002/bit.26197 27723092

[B77] Johnson B. A. Hage A. Kalveram B. Mears M. Plante J. A. Rodriguez S. E.. (2019). Peptidoglycan-associated cyclic lipopeptide disrupts viral infectivity. J. Virol. 93:e01282–e01219. 10.1128/JVI.01282-19 31462558 PMC6819921

[B78] Joshi S. J. Al-Wahaibi Y. M. Al-Bahry S. N. Elshafie A. E. Al-Bemani A. S. Al-Bahri A.. (2016). Production, characterization, and application of bacillus licheniformis W16 biosurfactant in enhancing oil recovery. Front. Microbiol. 7:1853. 10.3389/fmicb.2016.01853 27933041 PMC5120096

[B79] Jung J. Yu K. O. Ramzi A. B. Choe S. H. Kim S. W. Han S. O. (2012). Improvement of surfactin production in *Bacillus subtilis* using synthetic wastewater by overexpression of specific extracellular signaling peptides, *comX* and *phrC*. Biotechnol. Bioeng. 109, 2349–2356. 10.1002/bit.24524 22511326

[B80] Kakinuma A. Hori M. Isono M. Tamura G. Arima K. (1969a). Determination of amino acid sequence in surfactin, a crystalline peptidelipid surfactant produced by *Bacillus subtilis*. Agric. Biol. Chem. 33, 971–972. 10.1080/00021369.1969.10859408

[B81] Kakinuma A. Sugino H. Isono M. Tamura G. Arima K. (1969b). Determination of fatty acid in surfactin and elucidation of the total structure of surfactin. Agric. Biol. Chem. 33, 973–976. 10.1080/00021369.1969.10859409

[B82] Kämpfer P. (1994). Limits and possibilities of total fatty acid analysis for classification and identification of *Bacillus*species. Syst. Appl. Microbiol. 17, 86–98. 10.1016/S0723-2020(11)80035-4

[B83] Kaneda T. (1969). Fatty acids in *Bacillus larvae, Bacillus lentimorbus*, and *Bacillus popilliae*. J. Bacteriol. 98, 143–146. 10.1128/JB.98.1.143-146.1969 5781571 PMC249915

[B84] Kaneda T. (1991). Iso- and anteiso-fatty acids in bacteria: biosynthesis, function, and taxonomic significance. Microb. Rev. 55, 288–302. 10.1128/MR.55.2.288-302.1991 1886522 PMC372815

[B85] Kim H.-S. Yoon B.-D. Y. Suh H.-H. Oh H.-M. Katsuragi T. Tani Y. (1997). Production and properties of a lipopeptide biosurfactant from *Bacillus subtilis* C9. J. Ferment. Bioeng. 84, 41–46. 10.1016/S0922-338X(97)82784-5

[B86] Kowall M. Vater J. Kluge B. Stein T. Franke P. Ziessow D. (1998). Separation and characterization of surfactin isoforms produced by *Bacillus subtilis* OKB 105. J. Colloid Interface Sci. 204, 1–8. 10.1006/jcis.1998.5558 9665760

[B87] Kraas F. I. Helmetag V. Wittmann M. Strieker M. Marahiel M. A. (2010). Functional dissection of surfactin synthetase initiation module reveals insights into the mechanism of lipoinitiation. Chem. Biol. 17, 872–880. 10.1016/j.chembiol.2010.06.015 20797616

[B88] Kracht M. R. O. K. O. S. H. Ozel M. Kowall M. Pauli G. Vatera J. Özel M.. (1999). Antiviral and hemolytic activities of surfactin isoforms and their methyl ester derivatives. J. Antibiot. 52, 613–619. 10.7164/antibiotics.52.613 10513840

[B89] Lebecque S. Crowet J. M. Nasir M. N. Deleu M. Lins L. (2017). Molecular dynamics study of micelles properties according to their size. J. Mol. Graph. Model. 72, 6–15. 10.1016/j.jmgm.2016.12.007 27992815

[B90] Li X. Yang H. Zhang D. Li X. Yu H. Shen Z. (2015). Overexpression of specific proton motive force-dependent transporters facilitate the export of surfactin in *Bacillus subtilis*. J. Ind. Microbiol. Biotechnol. 42, 93–103. 10.1007/s10295-014-1527-z 25366377

[B91] Li Y. Yang S. Mu B. (2010). The surfactin and lichenysin isoforms produced by *Bacillus licheniformis* HSN 221. Anal. Lett. 43, 929–940. 10.1080/00032710903491047

[B92] Lien Grosdidier A. Zoete V. Michielin O. (2011). SwissDock, a protein-small molecule docking web service based on EADock DSS. Nucleic Acids Res. 39, 270–277. 10.1093/nar/gkr366 21624888 PMC3125772

[B93] Lins L. Brasseur R. (1995). The hydrophobic effect in protein folding. FASEB J. 9, 535–540. 10.1096/fasebj.9.7.7737462 7737462

[B94] Liu J. Zuber P. (1998). A molecular switch controlling competence and motility: competence regulatory factors ComS, MecA, and ComK control σ(D)-dependent gene expression in *Bacillus subtilis*. J. Bacteriol. 180, 4243–4251. 10.1128/JB.180.16.4243-4251.1998 9696775 PMC107423

[B95] Liu J. F. Yang J. Yang S. Z. Ye R. Q. Mu B. Z. (2012). Effects of different amino acids in culture media on surfactin variants produced by *Bacillus subtilis* TD7. Appl. Biochem. Biotechnol. 166, 2091–2100. 10.1007/s12010-012-9636-5 22415784

[B96] Liu K. Sun Y. Cao M. Wang J. Lu J. R. Xu H. (2020). Rational design, properties, and applications of biosurfactants: a short review of recent advances. Curr. Opin. Colloid Interface Sci. 45, 57–67. 10.1016/j.cocis.2019.12.005

[B97] Liu Q. Lin J. Wang W. Huang H. Li S. (2015). Production of surfactin isoforms by *Bacillus subtilis* BS-37 and its applicability to enhanced oil recovery under laboratory conditions. Biochem. Eng. J. 93, 31–37. 10.1016/j.bej.2014.08.023

[B98] Liu T. Montastruc L. Gancel F. Zhao L. Nikov I. (2007). Integrated process for production of surfactin. Part 1: adsorption rate of pure surfactin onto activated carbon. Biochem. Eng. J. 35, 333–340. 10.1016/j.bej.2007.01.025

[B99] Liu X. Y. Yang S. Z. Mu B. Z. (2009). Production and characterization of a C15-surfactin-O-methyl ester by a lipopeptide producing strain *Bacillus subtilis* HSO121. Process Biochem. 44, 1144–1151. 10.1016/j.procbio.2009.06.014

[B100] Loiseau C. Schlusselhuber M. Bigot R. Bertaux J. Berjeaud J. M. Verdon J. (2015). Surfactin from *Bacillus subtilis* displays an unexpected anti-*Legionella* activity. Appl. Microbiol. Biotechnol. 99, 5083–5093. 10.1007/s00253-014-6317-z 25573468

[B101] Long X. He N. He Y. Jiang J. Wu T. (2017). Biosurfactant surfactin with pH-regulated emulsification activity for efficient oil separation when used as emulsifier. Bioresour. Technol. 241, 200–206. 10.1016/j.biortech.2017.05.120 28570884

[B102] López D. Vlamakis H. Losick R. Kolter R. (2009). Paracrine signaling in a bacterium. Genes Dev. 23, 1631–1638. 10.1101/gad.1813709 19605685 PMC2714712

[B103] Lu Y. J. Zhang Y. M. Rock C. O. (2004). Product diversity and regulation of type II fatty acid synthases. Biochem. Cell Biol. 82, 145–155. 10.1139/o03-076 15052334

[B104] Maass D. Moya Ramírez I. García Román M. Jurado Alameda E. Ulson de Souza A. A. Borges Valle J. A.. (2016). Two-phase olive mill waste (alpeorujo) as carbon source for biosurfactant production. J. Chem. Technol. Biotechnol. 91, 1990–1997. 10.1002/jctb.4790

[B105] Maget-Dana R. Thimon L. Peypoux F. Ptak M. (1992). Surfactin/iturin A interactions may explain the synergistic effect of surfactin on the biological properties of iturin A. Biochimie 74, 1047–1051. 10.1016/0300-9084(92)90002-V 1292612

[B106] Marahiel M. A. Stachelhaus T. Mootz H. D. (1997). Modular peptide synthetases involved in nonribosomal peptide synthesis. Chem. Rev. 97, 2651–2673. 10.1021/cr960029e 11851476

[B107] Marcelino L. Puppin-Rontani J. Coutte F. Machini M. T. Etchegaray A. Puppin-Rontani R. M. (2019). Surfactin application for a short period (10/20 s) increases the surface wettability of sound dentin. Amino Acids 51, 1233–1240. 10.1007/s00726-019-02750-1 31197572

[B108] Marini P. Li S. J. Gardiol D. Cronan J. E. De Mendoza D. (1995). The genes encoding the biotin carboxyl carrier protein and biotin carboxylase subunits of *Bacillus subtilis* acetyl coenzyme a carboxylase, the first enzyme of fatty acid synthesis. J. Bacteriol. 177, 7003–7006. 10.1128/JB.177.23.7003-7006.1995 7592499 PMC177574

[B109] Marrink S. J. Risselada H. J. Yefimov S. Tieleman D. P. De Vries A. H. (2007). The MARTINI force field: coarse grained model for biomolecular simulations. J. Phys. Chem. B 111, 7812–7824. 10.1021/jp071097f 17569554

[B110] Martin N. Christensen Q. H. Mansilla M. C. Cronan J. E. de Mendoza D. (2011). A novel two-gene requirement for the octanoyltransfer reaction of *Bacillus subtilis* lipoic acid biosynthesis. Mol Microbiol. 80, 335–349. 10.1111/j.1365-2958.2011.07597.x 21338420 PMC3086205

[B111] Martinez L. Andrade R. Birgin E. G. Martínez J. M. (2009). PACKMOL: A package for building initial configurations for molecular dynamics simulations. J. Comput. Chem. 30, 2157–2164. 10.1002/jcc.21224 19229944

[B112] Matsunaga I. Ueda A. Fujiwara N. Sumimoto T. Ichihara K. (1999). Characterization of the *ybdT* gene product of *Bacillus subtilis*: novel fatty acid β-hydroxylating cytochrome P450. Lipids 34, 841–846. 10.1007/s11745-999-0431-3 10529095

[B113] Meena K. R. Parmar A. Sharma A. Kanwar S. S. (2018). A novel approach for body weight management using a bacterial surfactin lipopeptide. Obes. Med. 10, 24–28. 10.1016/j.obmed.2018.05.003

[B114] Menkhaus M. Ullrich C. Kluge B. Vater J. Vollenbroich D. Kamp R. M. (1993). Structural and functional organization of the surfactin synthetase multienzyme system. J. Biol. Chem. 268, 7678–7684. 10.1016/S0021-9258(18)53010-6 8096516

[B115] Miyazaki N. Sugai Y. Sasaki K. Okamoto Y. Yanagisawa S. (2020). Screening of the effective additive to inhibit surfactin from forming precipitation with divalent cations for surfactin enhanced oil recovery. Energies 13:2430. 10.3390/en13102430

[B116] Mnif I. Besbes S. Ellouze-Ghorbel R. Ellouze-Chaabouni S. Ghribi D. (2013). Improvement of bread dough quality by *Bacillus subtilis* SPB1 biosurfactant addition: optimized extraction using response surface methodology. J. Sci. Food Agric. 93, 3055–3064. 10.1002/jsfa.6139 23512731

[B117] Mohammadipour M. Mousivand M. Jouzani G. S. Abbasalizadeh S. (2009). Molecular and biochemical characterization of Iranian surfactin-producing *Bacillus subtilis* isolates and evaluation of their biocontrol potential against *Aspergillus flavus* and *Colletotrichum gloeosporioides*. Can. J. Microbiol. 55, 395–404. 10.1139/W08-141 19396239

[B118] Mootz H. D. Finking R. Marahiel M. A. (2001). 4′-Phosphopantetheine transfer in primary and secondary metabolism of *Bacillus subtilis*. J. Biol. Chem. 276, 37289–37298. 10.1074/jbc.M103556200 11489886

[B119] Mootz H. D. Kessler N. Linne U. Eppelmann K. Schwarzer D. Marahiel M. A. (2002). Decreasing the ring size of a cyclic nonribosomal peptide antibiotic by in-frame module deletion in the biosynthetic genes. J. Am. Chem. Soc. 124, 10980–10981. 10.1021/ja027276m 12224936

[B120] Morbidoni H. R. De Mendoza D. Cronan J. E. (1996). *Bacillus subtilis* acyl carrier protein is encoded in a cluster of lipid biosynthesis genes. J. Bacteriol. 178, 4794–4800. 10.1128/JB.178.16.4794-4800.1996 8759840 PMC178259

[B121] Morikawa M. Hirata Y. Imanaka T. (2000). A study on the structure-function relationship of lipopeptide biosurfactants. Biochim. Biophys. Acta - Gen. Subj. (1488). 211–218. 10.1016/S1388-1981(00)00124-4 11082531

[B122] Morris G. M. Ruth H. Lindstrom W. Sanner M. F. Belew R. K. Goodsell D. S.. (2009). Software news and updates AutoDock4 and AutoDockTools4: automated docking with selective receptor flexibility. J. Comput. Chem. 30, 2785–2791. 10.1002/jcc.21256 19399780 PMC2760638

[B123] Moya Ramírez I. Tsaousi K. Rudden M. Marchant R. Jurado Alameda E. García Román M.. (2015). Rhamnolipid and surfactin production from olive oil mill waste as sole carbon source. Bioresour. Technol. 198, 231–236. 10.1016/j.biortech.2015.09.012 26398666

[B124] Mulligan C. N. Yong R. N. Gibbs B. F. James S. Bennett H. P. J. (1999). Metal removal from contaminated soil and sediments by the biosurfactant surfactin. Environ. Sci. Technol. 33, 3812–3820. 10.1021/es9813055

[B125] Nagai S. Okimura K. Kaizawa N. Ohki K. Kanatomo S. (1996). Study on surfactin, a cyclic depsipeptide. II. Synthesis of surfactin B2 produced by *Bacillus natto* KMD (2311). Chem. Pharm. Bull. 44, 5–10. 10.1248/cpb.44.5 8582044

[B126] Nakano M. M. Corbell N. Besson J. Zuber P. (1992). Isolation and characterization of *sfp:* a gene that functions in the production of the lipopeptide biosurfactant, surfactin, in *Bacillus subtilis*. MGG Mol. Gen. Genet. 232, 313–321. 10.1007/BF00280011 1557038

[B127] Nakano M. M. Marahiel M. A. Zuber P. (1988). Identification of a genetic locus required for biosynthesis of the lipopeptide antibiotic surfactin in *Bacillus subtilis*. J. Bacteriol. 170, 5662–5668. 10.1128/JB.170.12.5662-5668.1988 2848009 PMC211666

[B128] Naruse N. Tenmyo O. Kobaru S. Kamei H. Miyaki T. Konishi M.. (1990). Pumilacidin, a complex of new antiviral antibiotics production, isolation, chemical properties, structure and biological activity. J. Antibiot. 43, 267–280. 10.7164/antibiotics.43.267 2157695

[B129] Niehren J. Versari C. John M. Coutte F. Jacques P. Predicting P. J. (2016). Predicting changes of reaction networks with partial kinetic information. BioSystems 149, 113–124. 10.1016/j.biosystems.2016.09.003 27769750

[B130] Nitschke M. Pastore G. M. (2004). Biosurfactant production by *Bacillus subtilis* using cassava-processing effluent. Appl. Biochem. Biotechnol. 112, 163–172. 10.1385/ABAB:112:3:163 15007184

[B131] Ohadi M. Shahravan A. Dehghannoudeh N. Eslaminejad T. Banat I. M. Dehghannoudeh G. (2020). Potential use of microbial surfactant in microemulsion drug delivery system: a systematic review. Drug Des. Devel. Ther. 14, 541–550. 10.2147/DDDT.S232325 32103896 PMC7008186

[B132] Ohno A. Ano T. Shoda M. (1995a). Effect of temperature on production of lipopeptide antibiotics, iturin A and surfactin by a dual producer, *Bacillus subtilis* RB14, in solid-state fermentation. J. Ferment. Bioeng. 80, 517–519. 10.1016/0922-338X(96)80930-5 18623394

[B133] Ohno A. Ano T. Shoda M. (1995b). Production of a lipopeptide antibiotic, surfactin, by recombinant *Bacillus subtilis* in solid state fermentation. Biotechnol. Bioeng. 47, 209–214. 10.1002/bit.260470212 18623394

[B134] Ohsawa T. Tsukahara K. Sato T. Ogura M. (2006). Superoxide stress decreases expression of *srfA* through inhibition of transcription of the *comQXP* quorum-sensing locus in *Bacillus subtilis*. J. Biochem. 139, 203–211. 10.1093/jb/mvj023 16452308

[B135] Ong S. A. Wu J. C. (2018). A simple method for rapid screening of biosurfactant-producing strains using bromothymol blue alone. Biocatal. Agric. Biotechnol. 16, 121–125. 10.1016/j.bcab.2018.07.027

[B136] Ongena M. Jacques P. (2008). *Bacillus* lipopeptides: versatile weapons for plant disease biocontrol. Trends Microbiol. 16, 115–125. 10.1016/j.tim.2007.12.009 18289856

[B137] Ongena M. Jourdan E. Adam A. Paquot M. Brans A. Joris B.. (2007). Surfactin and fengycin lipopeptides of *Bacillus subtilis* as elicitors of induced systemic resistance in plants. Environ. Microbiol. 9, 1084–1090. 10.1111/j.1462-2920.2006.01202.x 17359279

[B138] Pagadoy M. Peypoux F. Wallach J. (2005). Solid-phase synthesis of surfactin, a powerful biosurfactant produced by *Bacillus subtilis*, and of four analogues. Int. J. Pept. Res. Ther. 11, 195–202. 10.1007/s10989-005-6790-4

[B139] Paraszkiewicz K. Bernat P. Kuśmierska A. Chojniak J. Płaza G. (2018). Structural identification of lipopeptide biosurfactants produced by *Bacillus subtilis* strains grown on the media obtained from renewable natural resources. J. Environ. Manage. 209, 65–70. 10.1016/j.jenvman.2017.12.033 29275286

[B140] Park S. Y. Kim Y. H. (2009). Surfactin inhibits immunostimulatory function of macrophages through blocking NK-κB, MAPK and Akt pathway. Int. Immunopharmacol. 9, 886–893. 10.1016/j.intimp.2009.03.013 19336264

[B141] Peypoux F. Bonmatin J. Labbe H. Grangemard I. Das B. C. Ptak M.. (1994). [Ala4]Surfactin, a novel isoform from. Eur. J. Biochem. 224, 89–96. 10.1111/j.1432-1033.1994.tb19998.x 8076655

[B142] Peypoux F. Bonmatin J.-M. Labbé H. Das B. C. Ptak M. Michel G. (1991). Isolation and characterization of a new variant of surfactin, the [Val7]surfactin. Eur. J. Biochem. 202, 101–106. 10.1111/j.1432-1033.1991.tb16349.x 1935967

[B143] Quadri L. E. N. Weinreb P. H. Lei M. Nakano M. M. Zuber P. Walsh C. T. (1998). Characterization of Sfp, a *Bacillus subtilis* phosphopantetheinyl transferase for peptidyl carder protein domains in peptide synthetases. Biochemistry 37, 1585–1595. 10.1021/bi9719861 9484229

[B144] Rausch C. Hoof I. Weber T. Wohlleben W. Huson D. H. (2007). Phylogenetic analysis of condensation domains in NRPS sheds light on their functional evolution. BMC Evol. Biol. 7:78. 10.1186/1471-2148-7-78 17506888 PMC1894796

[B145] Rausch C. Weber T. Kohlbacher O. Wohlleben W. Huson D. H. (2005). Specificity prediction of adenylation domains in nonribosomal peptide synthetases (NRPS) using transductive support vector machines (TSVMs). Nucleic Acids Res. 33, 5799–5808. 10.1093/nar/gki885 16221976 PMC1253831

[B146] Razafindralambo H. Popineau Y. Deleu M. Hbid C. Jacques P. Thonart P.. (1998). Foaming properties of lipopeptides produced by *Bacillus subtilis*: effect of lipid and peptide structural attributes. J. Agric. Food Chem. 46, 911–916. 10.1021/jf970592d

[B147] Roongsawang N. Washio K. Morikawa M. (2011). Diversity of nonribosomal peptide synthetases involved in the biosynthesis of lipopeptide biosurfactants. Int. J. Mol. Sci. 12, 141–172. 10.3390/ijms12010141 21339982 PMC3039948

[B148] Runguphan W. Keasling J. D. (2014). Metabolic engineering of *Saccharomyces cerevisiae* for production of fatty acid-derived biofuels and chemicals. Metab. Eng. 21, 103–113. 10.1016/j.ymben.2013.07.003 23899824

[B149] Schneider A. Stachelhaus T. Marahiel M. A. (1998). Targeted alteration of the substrate specificity of peptide synthetases by rational module swapping. Mol. Gen. Genet. 257, 308–318. 10.1007/s004380050652 9520265

[B150] Schneider K. B. Palmer T. M. Grossman A. D. (2002). Characterization of *comQ* and *comX*, two genes required for production of comX pheromone in *Bacillus subtilis*. J. Bacteriol. 184, 410–419. 10.1128/JB.184.2.410-419.2002 11751817 PMC139578

[B151] Schujman G. E. Choi K. Altabe S. Rock C. O. de Mendoza D. (2001). Response of *Bacillus subtilis*to cerulenin and acquisition of resistance. J. Bacteriol. 183, 3032–3040. 10.1128/JB.183.10.3032-3040.2001 11325930 PMC95202

[B152] Schwarzer D. Mootz H. D. Linne U. Marahiel M. A. (2002). Regeneration of misprimed nonribosomal peptide synthetases by type II thioesterases. Proc. Natl. Acad. Sci. U.S.A. 99, 14083–14088. 10.1073/pnas.212382199 12384573 PMC137840

[B153] Serre L. Swenson L. Green R. Wei Y. Verwoert I.ra, I. G. S. . (1994). Crystallization of the malonyl coenzyme A-acyl carrier protein transacylase from *Escherichia coli*. J Mol Biol. 242, 99–102. 10.1006/jmbi.1994.1559 8078074

[B154] Serre L. Verbree E. C. Dauter Z. Stuitje A. R. Derewenda Z. S. (1995). The *Escherichia coli* malonyl-CoA:acyl carrier protein transacylase at 1.5-A resolution. Crystal structure of a fatty acid synthase component. J. Biol. Chem. 270, 12961–12964. 10.1074/jbc.270.22.12961 7768883

[B155] Shakerifard P. Gancel F. Jacques P. Faille C. (2009). Effect of different *Bacillus subtilis* lipopeptides on surface hydrophobicity and adhesion of *Bacillus cereus* 98/4 spores to stainless steel and Teflon. Biofouling 25, 533–541. 10.1080/08927010902977943 19431000

[B156] Shao C. Liu L. Gang H. Yang S. Mu B. (2015). Structural diversity of the microbial surfactin derivatives from selective esterification approach. Int. J. Mol. Sci. 16, 1855–1872. 10.3390/ijms16011855 25599527 PMC4307338

[B157] Sieber S. A. Marahiel M. A. (2005). Molecular mechanisms underlying nonribosomal peptide synthesis: approaches to new antibiotics. Chem. Rev. 105, 715–738. 10.1021/cr0301191 15700962

[B158] Sinderen D. Galli G. Cosmina P. Ferra F. Withoff S. Venema G.. (1993). Characterization of the srfA locus of *Bacillus subtilis*: only the valine-activating domain of srfA is involved in the establishment of genetic competence. Mol. Microbiol. 8, 833–841. 10.1111/j.1365-2958.1993.tb01630.x 8355610

[B159] Smyth T. J. Perfumo A. Mcclean S. Banat I. M. (2010). “Isolation and analysis of lipopeptides and high molecular weight biosurfactants,” in Handbook of Hydrocarbon and Lipid Microbiology, eds K. N. Timmis, T. J. McGenity, J. R. vanderMeer, and V. deLorenzo (Berlin; Heidelberg: Springer), 3689–3704. 10.1007/978-3-540-77587-4_290

[B160] Stachelhaus T. Schneider A. Marahiel M. A. (1995). Rational design of peptide antibiotics by targeted replacement of bacterial and fungal domains. Science 269, 69–72. 10.1126/science.7604280 7604280

[B161] Stachelhaus T. Schneider A. Marahiel M. A. (1996). Engineered biosynthesis of peptide antibiotics. Biochem. Pharmacol. 52, 177–186. 10.1016/0006-2952(96)00111-6 8694841

[B162] Steen E. J. Kang Y. Bokinsky G. Hu Z. Schirmer A. McClure A.. (2010). Microbial production of fatty-acid-derived fuels and chemicals from plant biomass. Nature 463, 559–562. 10.1038/nature08721 20111002

[B163] Steller S. Sokoll A. Wilde C. Bernhard F. Franke P. Vater J. (2004). Initiation of surfactin biosynthesis and the role of the SrfD-thioesterase protein. Biochemistry 43, 11331–11343. 10.1021/bi0493416 15366943

[B164] Stiegelmeyer S. M. Giddings M. C. (2013). Agent-based modeling of competence phenotype switching in Bacillus subtilis. Theor. Biol. Med. Model. 10:23. 10.1186/1742-4682-10-23 23551850 PMC3648451

[B165] Süssmuth R. D. Mainz A. (2017). Nonribosomal peptide synthesis—principles and prospects. Angew. Chemie - Int. Ed. 56, 3770–3821. 10.1002/anie.201609079 28323366

[B166] Taira T. Yanagisawa S. Nagano T. Tsuji T. Endo A. Imura T. (2017). pH-induced conformational change of natural cyclic lipopeptide surfactin and the effect on protease activity. Colloids Surfaces B Biointerf. 156, 382–387. 10.1016/j.colsurfb.2017.05.017 28551572

[B167] Takahashi T. Ohno O. Ikeda Y. Sawa R. Homma Y. Igarashi M.. (2006). Inhibition of lipopolysaccharide activity by a bacterial cyclic lipopeptide surfactin. J. Antibiot. 59, 35–43. 10.1038/ja.2006.6 16568717

[B168] Tanaka K. Amaki Y. Ishihara A. Nakajima H. (2015). Synergistic effects of [Ile7]Surfactin homologues with bacillomycin d in suppression of gray mold disease by *Bacillus amyloliquefaciens* biocontrol strain SD-32. J. Agric. Food Chem. 63, 5344–5353. 10.1021/acs.jafc.5b01198 25976169

[B169] Tanaka K. Henry C. S. Zinner J. F. Jolivet E. Cohoon M. P. Xia F.. (2013). Building the repertoire of dispensable chromosome regions in *Bacillus subtilis* entails major refinement of cognate large-scale metabolic model. Nucleic Acids Res. 41, 687–699. 10.1093/nar/gks963 23109554 PMC3592452

[B170] Tang J.-S. Gao H. Hong K. Yu W. Jiang M.-M. Lin H.-P.. (2007). Complete assignement of 1H and 13C NMR spectral data of nine surfactin isomers. Magn. Reson. Chem. 45, 488–495. 10.1002/mrc.2048 17640005

[B171] Tong L. (2013). Structure and function of biotin-dependent carboxylases. Cell. Mol. Life Sci. 70, 863–891. 10.1007/s00018-012-1096-0 22869039 PMC3508090

[B172] Trauger J. W. Kohli R. M. Mootz H. D. Marahiel M. A. Walsh C. T. (2000). Peptide cyclization catalysed by the thioesterase domain of tyrocidine synthetase. Nature 407, 215–218. 10.1038/35025116 11001063

[B173] Tsuge K. Ano T. Hirai M. Nakamura Y. Shoda M. (1999). The genes *degQ, pps*, and *lpa-8 (sfp)* are responsible for conversion of *Bacillus subtilis* 168 to plipastatin production. Antimicrob. Agents Chemother. 43, 2183–2192. 10.1128/AAC.43.9.2183 10471562 PMC89444

[B174] Tsuge K. Ohata Y. Shoda M. (2001). Gene *yerP*, involved in surfactin self-resistance in *Bacillus subtilis*. Antimicrob. Agents Chemother. 45, 3566–3573. 10.1128/AAC.45.12.3566-3573.2001 11709341 PMC90870

[B175] Varvaresou A. Iakovou K. (2015). Biosurfactants in cosmetics and biopharmaceuticals. Lett. Appl. Microbiol. 61, 214–223. 10.1111/lam.12440 25970073

[B176] Verma A. Kumar A. Debnath M. (2016). Molecular docking and simulation studies to give insight of surfactin amyloid interaction for destabilizing Alzheimer's Ab42 protofibrils. Med. Chem. Res. 25, 1616–1622. 10.1007/s00044-016-1594-y

[B177] Vollenbroich D. Pauli G. Ozel M. Vater J. (1997). Antimycoplasma properties and application in cell culture of surfactin, a lipopeptide antibiotic from Bacillus subtilis. Appl. Environ. Microbiol. 63, 44–49. 10.1128/AEM.63.1.44-49.1997 8979337 PMC168300

[B178] Wakil S. J. Stoops J. K. Joshi V. C. (1983). Fatty acid synthesis and its regulation. Annu. Rev. Biochem. 52, 537–579. 10.1146/annurev.bi.52.070183.002541 6137188

[B179] Wang C. Cao Y. Wang Y. Sun L. Song H. (2019). Enhancing surfactin production by using systematic CRISPRi repression to screen amino acid biosynthesis genes in Bacillus subtilis. Microb. Cell Fact. 18:90. 10.1186/s12934-019-1139-4 31122258 PMC6533722

[B180] Wang X. Cai T. Wen W. Ai J. Ai J. Zhang Z.. (2020). Surfactin for enhanced removal of aromatic hydrocarbons during biodegradation of crude oil. Fuel 267:117272. 10.1016/j.fuel.2020.117272

[B181] Wei Y. H. Lai C. C. Chang J. S. (2007). Using Taguchi experimental design methods to optimize trace element composition for enhanced surfactin production by *Bacillus subtilis* ATCC 21332. Process Biochem. 42, 40–45. 10.1016/j.procbio.2006.07.025

[B182] Willecke K. Pardee A. B. (1971). Fatty acid-requiring mutant of *Bacillus subtilis* defective in branched chain alpha-keto acid dehydrogenase. J. Biol. Chem. 246, 5264–5272. 10.1016/S0021-9258(18)61902-7 4999353

[B183] Willenbacher J. Mohr T. Henkel M. Gebhard S. Mascher T. Syldatk C.. (2016). Substitution of the native srfA promoter by constitutive Pvegin two *B. Subtilis* strains and evaluation of the effect on Surfactin production. J. Biotechnol. 224, 14–17. 10.1016/j.jbiotec.2016.03.002 26953743

[B184] Willenbacher J. Yeremchuk W. Mohr T. Syldatk C. Hausmann R. (2015). Enhancement of Surfactin yield by improving the medium composition and fermentation process. AMB Express 5:57. 10.1186/s13568-015-0145-0 26297438 PMC4546119

[B185] Willenbacher J. Zwick M. Mohr T. Schmid F. Syldatk C. Hausmann R. (2014). Evaluation of different *Bacillus* strains in respect of their ability to produce Surfactin in a model fermentation process with integrated foam fractionation. Appl. Microbiol. Biotechnol. 98, 9623–9632. 10.1007/s00253-014-6010-2 25158834

[B186] Wu Q. Zhi Y. Xu Y. (2019). Systematically engineering the biosynthesis of a green biosurfactant surfactin by Bacillus subtilis 168. Metab. Eng. 52, 87–97. 10.1016/j.ymben.2018.11.004 30453038

[B187] Wu Y. S. Ngai S. C. Goh B. H. Chan K. G. Lee L. H. Chuah L. H. (2017). Anticancer activities of surfactin potential application of nanotechnology assisted surfactin delivery. Front. Pharmacol. 8:761. 10.3389/fphar.2017.00761 29123482 PMC5662584

[B188] Yakimov M. M. Abraham W. R. Meyer H. Laura G. Golyshin P. N. (1999). Structural characterization of lichenysin A components by fast atom bombardment tandem mass spectrometry. Biochim. Biophys. Acta 273–280. 10.1016/S1388-1981(99)00058-X 10320810

[B189] Yakimov M. M. Fredrickson H. L. Timmis K. N. (1996). Effect of heterogeneity of hydrophobic moieties on surface activity of lichenysin A, a lipopeptide biosurfactant from *Bacillus licheniformis* BASSO. Biotechnol. Appl. Biochem. 23, 13–18. 8867891

[B190] Yakimov M. M. Timmis K. N. Wray V. Fredrickson H. L. (1995). Characterization of a new lipopeptide surfactant produced by thermotolerant and halotolerant subsurface Bacillus licheniformis BAS50. Appl. Environ. Microbiol. 61, 1706–1713. 10.1128/AEM.61.5.1706-1713.1995 7646007 PMC167432

[B191] Yan X. Yu H. J. Hong Q. Li S. P. (2008). Cre/lox system and PCR-based genome engineering in *Bacillus subtilis*. Appl. Environ. Microbiol. 74, 5556–5562. 10.1128/AEM.01156-08 18641148 PMC2546623

[B192] Yang H. Li X. Li X. Yu H. Shen Z. (2015a). Identification of lipopeptide isoforms by MALDI-TOF-MS/MS based on the simultaneous purification of iturin, fengycin, and surfactin by RP-HPLC. Anal. Bioanal. Chem. 407, 2529–2542. 10.1007/s00216-015-8486-8 25662934

[B193] Yang H. Yu H. Shen Z. (2015b). A novel high-throughput and quantitative method based on visible color shifts for screening *Bacillus subtilis* THY-15 for surfactin production. J. Ind. Microbiol. Biotechnol. 42, 1139–1147. 10.1007/s10295-015-1635-4 26065390

[B194] Yang Z. Zu Y. Zhu J. Jin M. Cui T. Long X. (2020). Application of biosurfactant surfactin as a pH-switchable biodemulsifier for efficient oil recovery from waste crude oil. Chemosphere 240:124946. 10.1016/j.chemosphere.2019.124946 31726598

[B195] Yeh E. Kohli R. M. Bruner S. D. Walsh C. T. (2004). Type II thioesterase restores activity of a nrps module stalled with an aminoacyl-S-enzyme that cannot be elongated. ChemBioChem 5, 1290–1293. 10.1002/cbic.200400077 15368584

[B196] Yeh M. S. Wei Y. H. Chang J. S. (2005). Enhanced production of surfactin from *Bacillus subtilis* by addition of solid carriers. Biotechnol. Prog. 21, 1329–1334. 10.1021/bp050040c 16080719

[B197] Youssef N. H. Duncan K. E. Nagle D. P. Savage K. N. Knapp R. M. McInerney M. J. (2004). Comparison of methods to detect biosurfactant production by diverse microorganisms. J. Microbiol. Methods 56, 339–347. 10.1016/j.mimet.2003.11.001 14967225

[B198] Youssef N. H. Wofford N. McInerney M. J. (2011). Importance of the long-chain fatty acid beta-hydroxylating cytochrome P450 Enzyme YbdT for lipopeptide biosynthesis in *Bacillus subtilis* strain OKB105. Int. J. Mol. Sci. 12, 1767–1786. 10.3390/ijms12031767 21673922 PMC3111633

[B199] Yuan L. Zhang S. Wang Y. Li Y. Wang X. Yang Q. (2018). Surfactin inhibits membrane fusion during invasion of epithelial cells by enveloped viruses. J. Virol. 92, 1–19. 10.1128/JVI.00809-18 30068648 PMC6189506

[B200] Zanotto A. W. Valério A. de Andrade C. J. Pastore G. M. (2019). New sustainable alternatives to reduce the production costs for surfactin 50 years after the discovery. Appl. Microbiol. Biotechnol. 103, 8647–8656. 10.1007/s00253-019-10123-7 31515599

[B201] Zezzi do Valle Gomes M. Nitschke M. (2012). Evaluation of rhamnolipid and surfactin to reduce the adhesion and remove biofilms of individual and mixed cultures of food pathogenic bacteria. Food Control 25, 441–447. 10.1016/j.foodcont.2011.11.025

[B202] Zhang Y. Nakano S. Choi S. Y. Zuber P. (2006). Mutational analysis of the *Bacillus subtilis* RNA polymerase α C-terminal domain supports the interference model of Spx-dependent repression. J. Bacteriol. 188, 4300–4311. 10.1128/JB.00220-06 16740936 PMC1482945

[B203] Zhao H. Shao D. Jiang C. Shi J. Li Q. Huang Q.. (2017). Biological activity of lipopeptides from *Bacillus*. Appl. Microbiol. Biotechnol. 101, 5951–5960. 10.1007/s00253-017-8396-0 28685194

[B204] Zhuravleva O. I. Afiyatullov S. S. Ermakova S. P. Nedashkovskaya O. I. Dmitrenok P. S. Denisenko V. A.. (2010). New C14-surfactin methyl ester from the marine bacterium *Bacillus pumilus* KMM 456. Russ. Chem. Bull. 59, 2137–2142. 10.1007/s11172-010-0369-8

[B205] Zou A. Liu J. Garamus V. M. Yang Y. Willumeit R. Mu B. (2010). Micellization activity of the natural lipopeptide [Glu1, Asp5] surfactin-C15 in aqueous solution. J. Phys. Chem. B 114, 2712–2718. 10.1021/jp908675s 20146511

[B206] Zouari R. Besbes S. Ellouze-Chaabouni S. Ghribi-Aydi D. (2016). Cookies from composite wheat-sesame peels flours: dough quality and effect of *Bacillus subtilis* SPB1 biosurfactant addition. Food Chem. 194, 758–769. 10.1016/j.foodchem.2015.08.064 26471616

[B207] Zouboulis A. I. Matis K. A. Lazaridis N. K. Golyshin P. N. (2003). The use of biosurfactants in flotation: application for the removal of metal ions. Miner. Eng. 16, 1231–1236. 10.1016/j.mineng.2003.06.013

[B208] Zune Q. Telek S. Calvo S. Salmon T. Alchihab M. Toye D.. (2016). Influence of liquid phase hydrodynamics on biofilm formation on structured packing: optimization of surfactin production from Bacillus amyloliquefaciens. Chem. Eng. Sci. 170, 628–638. 10.1016/j.ces.2016.08.023

